# Supplementary figures and images for: Involvement of plasminogen activator inhibitor-1 in p300/p53-mediated age-related atrial fibrosis
Source: PeerJ. 2023 Dec 12;11:e16545. doi: 10.7717/peerj.16545 (PMC10722982; doi:10.7717/peerj.16545)

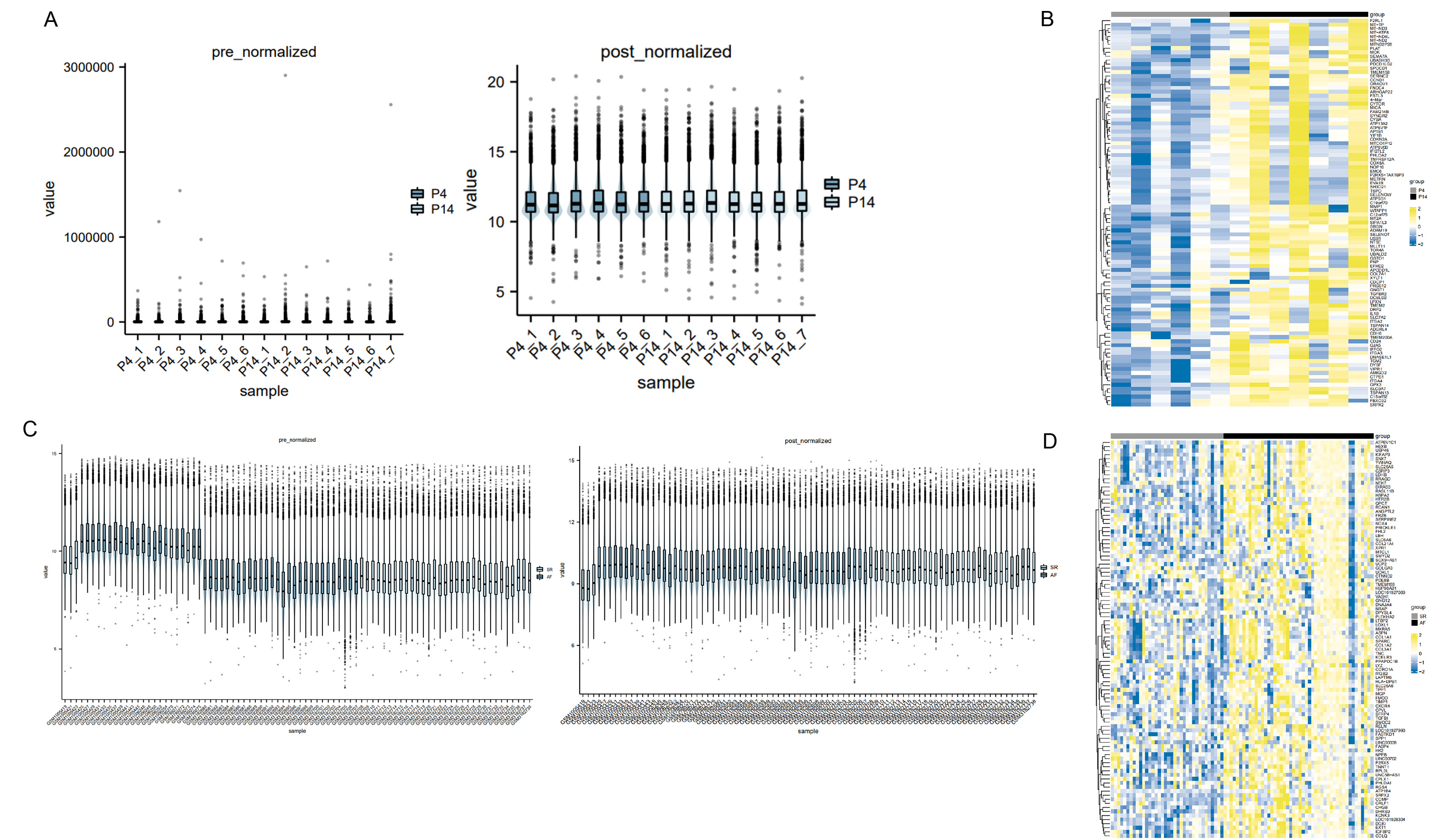

Supplement: Supplemental Information 4 — Heat maps of the differentially expressed genes are in (B) and (D). [file peerj-11-16545-s004.png]
