# Supplementary material for: Involvement of plasminogen activator inhibitor-1 in p300/p53-mediated age-related atrial fibrosis
Source: PeerJ. 2023 Dec 12;11:e16545. doi: 10.7717/peerj.16545 (PMC10722982; doi:10.7717/peerj.16545)
Supplement: Supplemental Information 5 [file peerj-11-16545-s005.doc]

**Supplement to Methods section**

**Antibodies used in this study.**

| Antibodies | Source |  |
| --- | --- | --- |
| Mouse anti-p300 (1:1000) | Millipore | Cat^#^05-257 |
| Mouse anti-p53 (1:1000) | Cell Signaling Technology | Cat^#^2524 |
| Mouse anti-p21 (1:1000) | Santa cruz | Cat^#^sc-817 |
| Rabbit anti-PAI-1 (1:1000) | Cell Signaling Technology | Cat^#^11907 |
| Rabbit anti-Collagen Ⅰ antibody (1:50) | Abcam | Cat^#^ab34710 |
| Mouse anti-GAPDH (1:10000) | Proteintech | Cat^#^60004-1-1g |
| AffiniPure Goat Anti-Mouse IgG (1:5000) | Jackson | Cat^#^115-035-003 |
| AffiniPure Goat Anti-Rabbit IgG (1:5000) | Jackson | Cat^#^111-035-003 |

For p300 knockdown of human atrial fibroblasts, the sequences are shown below:

Target sequences：

1. EP300-RNAi-1: ccC GGT GAA CTC TCC TAT AAT;
2. EP300-RNAi-2: ccA GCC TCA AAC TAC AAT AAA;
3. EP300-RNAi-3: cgA GTC TTC TTT CTG ACT CAA;

**shRNAs sequences:**

1）Ep300-RNAi-1-5’：GATCCCGCGGAATACTATCACCTCCTACTCGAGTAGGAGGTGATAGTATTCCGCTTTTTGGAT

2）Ep300-RNAi-1-3’：

GGCGCCTTATGATAGTGGAGGATGAGCTCATCCTCCACTATCATAAGGCGAAAAACCTATCGA

3）Ep300-RNAi-2-5’：

GATCCCGCTAGTCCTATGGGTGTAAATCTCGAGATTTACACCCATAGGACTAGCTTTTTGGAT

4）Ep300-RNAi-2-3’：

GGCGATCAGGATACCCACATTTAGAGCTCTAAATGTGGGTATCCTGATCGAAAAACCTATCGA

5）Ep300-RNAi-3-5’：GATCCCccAGTCCTTATGGTTCACCATCTCGAGATGGTGAACCATAAGGACTGGTTTTTGGAT

6）Ep300-RNAi-3-3’：

GGGGTCAGGAATACCAAGTGGTAGAGCTCTACCACTTGGTATTCCTGACCAAAAACCTATCGA

All the shRNAs were purchased from Shanghai Genechem Co.,Ltd, China.

siRNA specific for human p53:

1. hTP53 si-1 sense GCG CAC AGA GGA AGA GAA UTT;
2. hTP53 si-2 sense CCA CUG GAU GGA GAA UAU UTT;
3. hTP53 si-3 sense CCA UCC ACU ACA ACU ACA UTT.

siRNA specific for mouse p300:

1) EP300-RNAi-1: GCACGAACTAGGAAAGAAA;

2) EP300-RNAi-2: TTGGGACTAACCAATGGTG

3) EP300-RNAi-3: GCGGCCTAAACTCTCATCT

siRNA specific for mouse p53:

1. mTrp53 siRNA-1 sense:GAAUGAGGCCUUAGAGUUATT
2. mTrp53 siRNA-2 sense:AAGUCUGUUAUGUGCACGUACTT
3. mTrp53 siRNA-3 sense: CCACUUGAUGGAGAGUAUUTT

All the siRNAs were purchased from Shanghai Genechem Co.,Ltd, China
