# Supplementary material for: Involvement of plasminogen activator inhibitor-1 in p300/p53-mediated age-related atrial fibrosis
Source: PeerJ. 2023 Dec 12;11:e16545. doi: 10.7717/peerj.16545 (PMC10722982; doi:10.7717/peerj.16545)
Supplement: Supplemental Information 8 [file peerj-11-16545-s008.zip › Uncropped Gels/Uncropped Gels.pptx]

## Slide 1
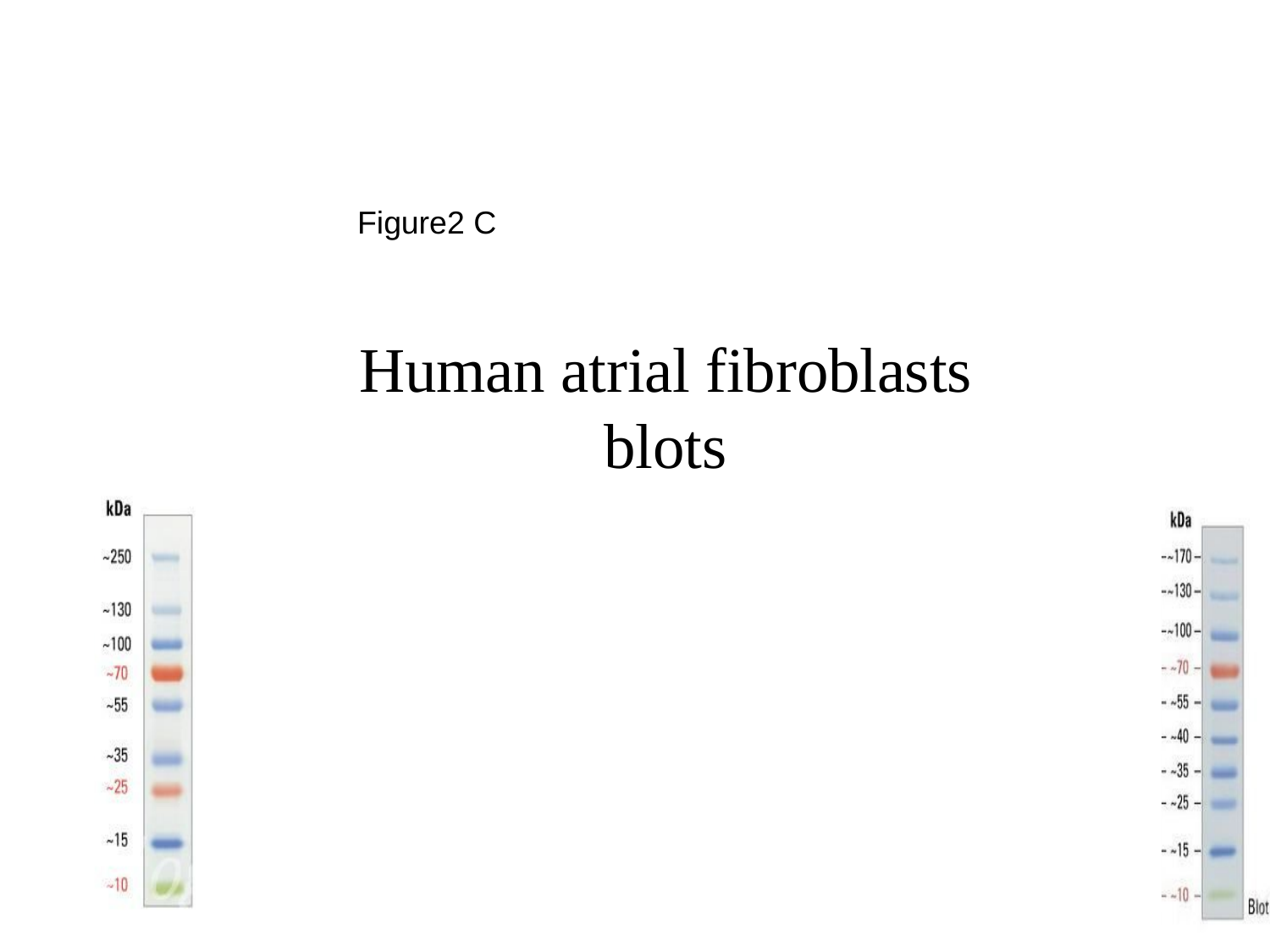

Figure2 C
# Human atrial fibroblasts blots

## Slide 2
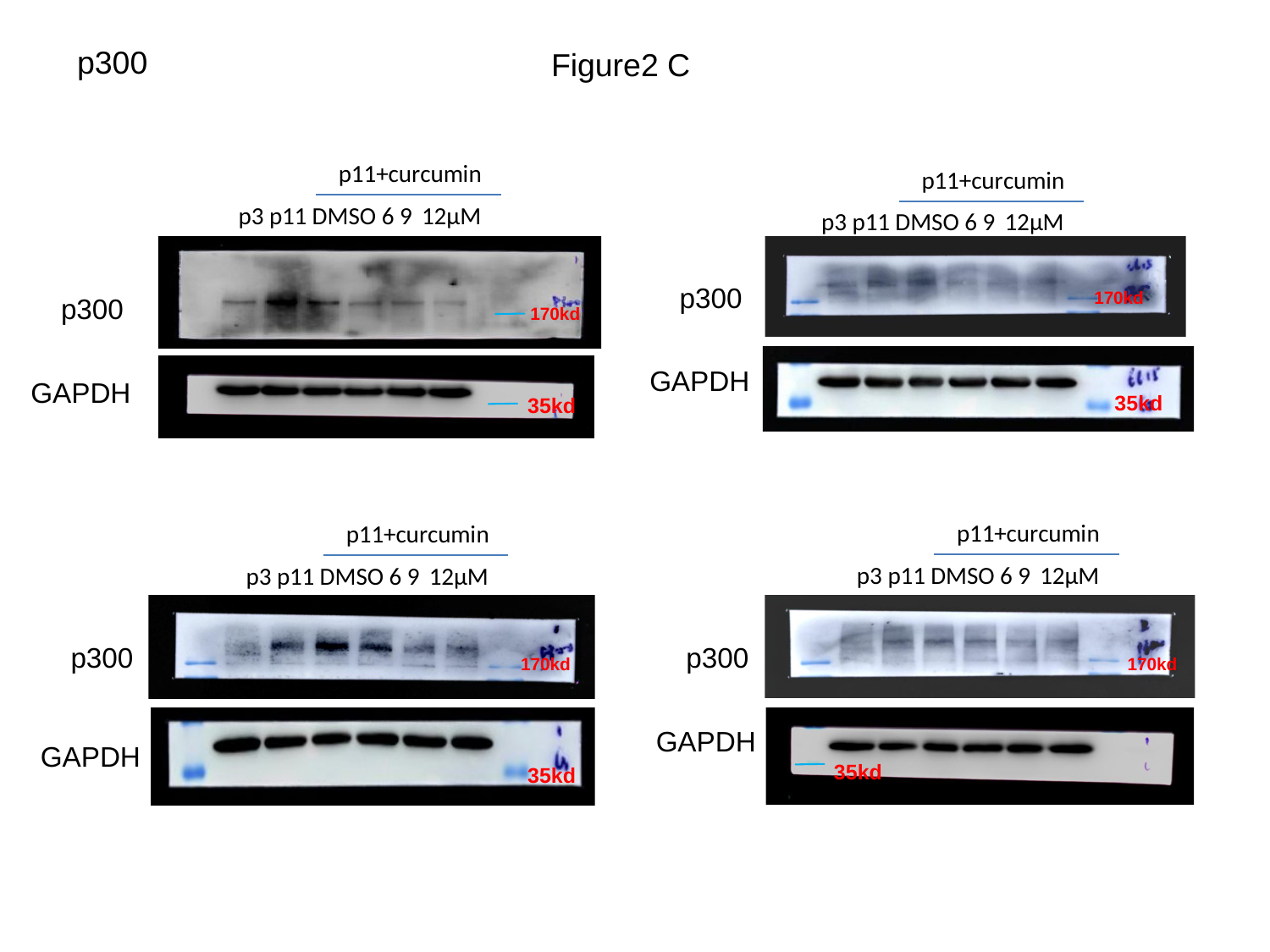

p300
Figure2 C
p11+curcumin
p11+curcumin
p3 p11 DMSO 6 9 12μM
p3 p11 DMSO 6 9 12μM
p300
170kd
p300
170kd
GAPDH
GAPDH
35kd
35kd
p11+curcumin
p11+curcumin
p3 p11 DMSO 6 9 12μM
p3 p11 DMSO 6 9 12μM
p300
p300
170kd
170kd
GAPDH
GAPDH
35kd
35kd

## Slide 3
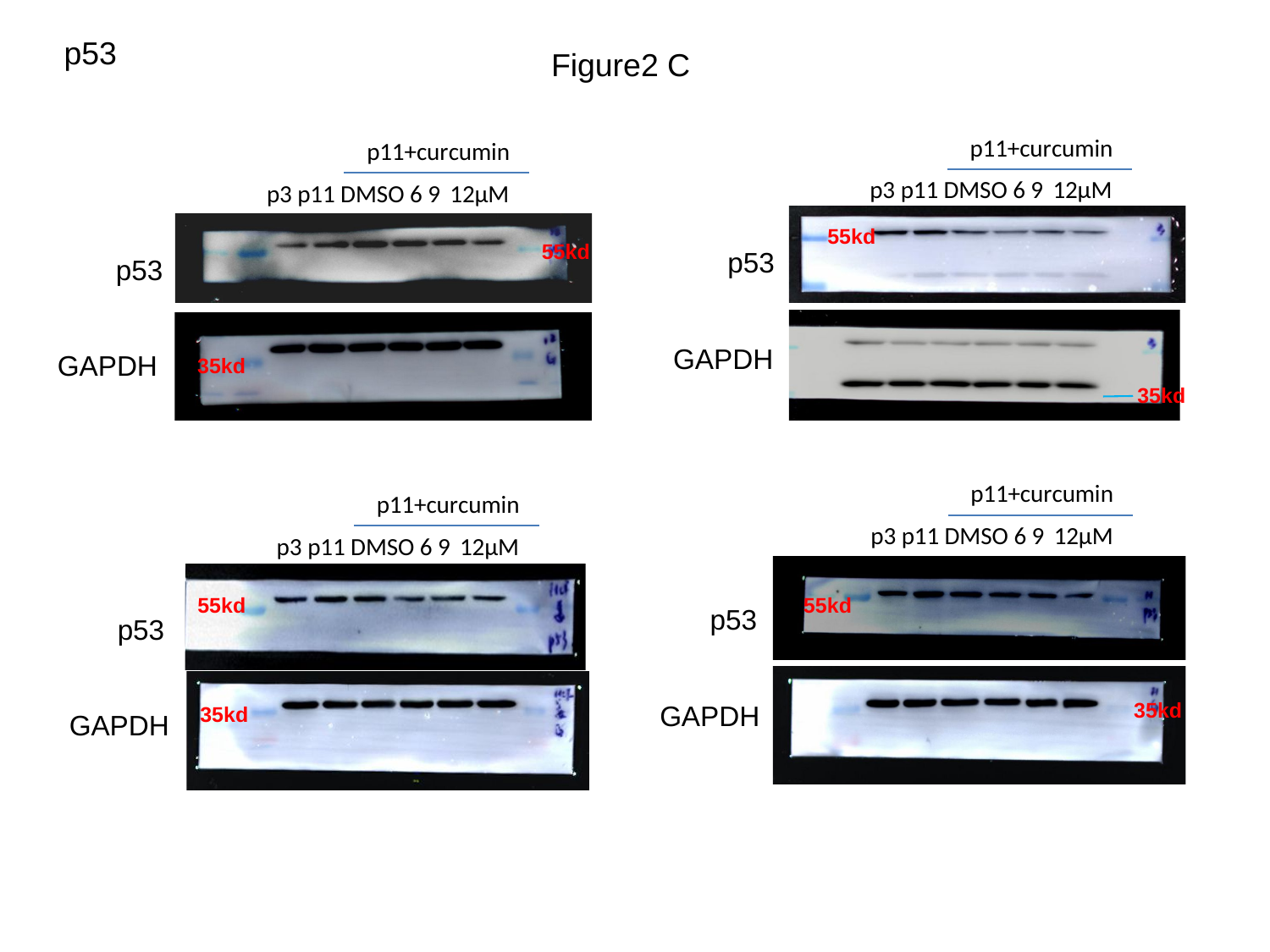

p53
Figure2 C
p11+curcumin
p11+curcumin
p3 p11 DMSO 6 9 12μM
p3 p11 DMSO 6 9 12μM
55kd
55kd
p53
p53
GAPDH
GAPDH
35kd
35kd
p11+curcumin
p11+curcumin
p3 p11 DMSO 6 9 12μM
p3 p11 DMSO 6 9 12μM
55kd
55kd
p53
p53
35kd
GAPDH
35kd
GAPDH

## Slide 4
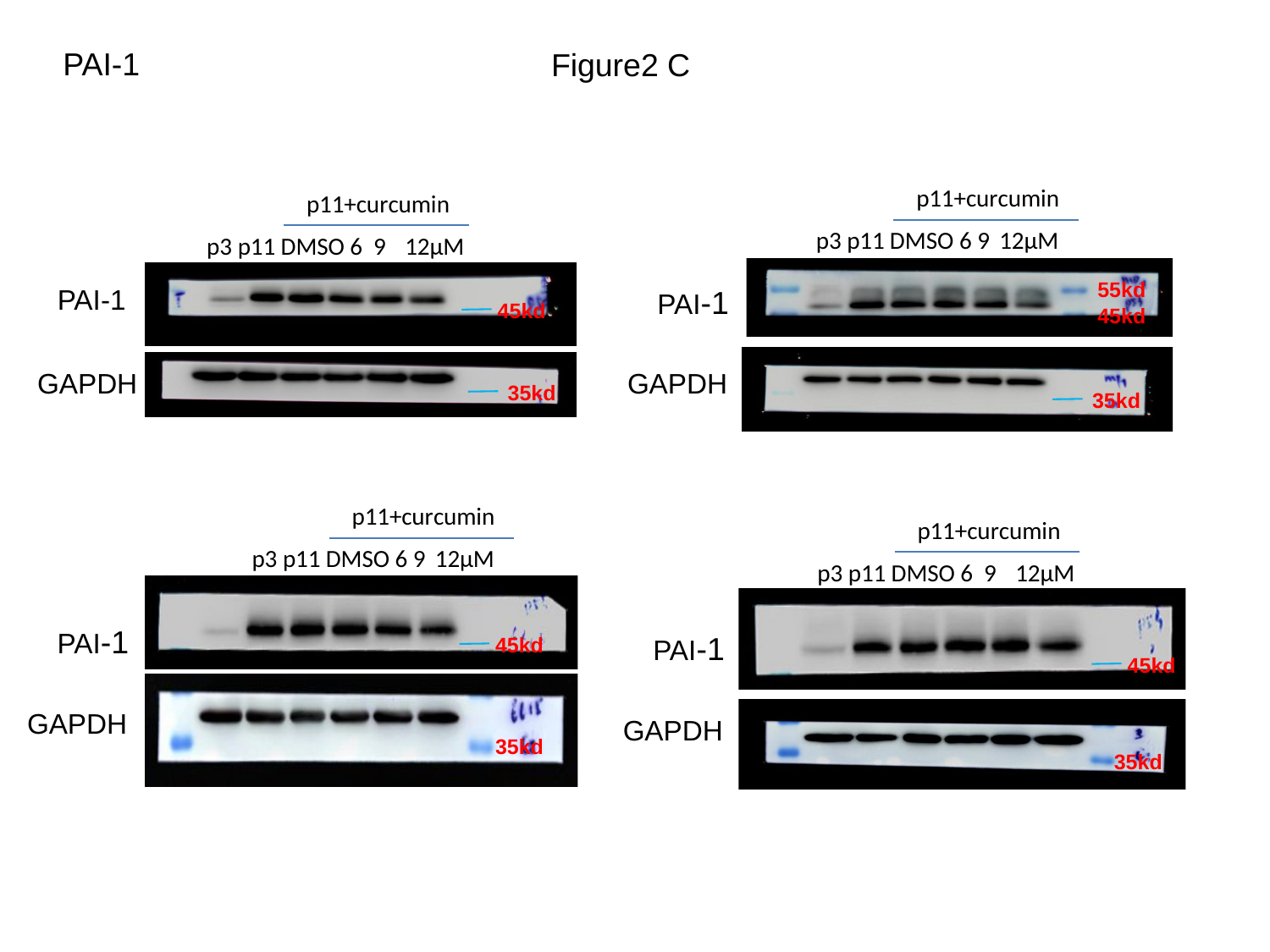

PAI-1
Figure2 C
p11+curcumin
p11+curcumin
p3 p11 DMSO 6 9 12μM
p3 p11 DMSO 6 9 12μM
55kd
PAI-1
PAI-1
45kd
45kd
GAPDH
GAPDH
35kd
35kd
p11+curcumin
p11+curcumin
p3 p11 DMSO 6 9 12μM
p3 p11 DMSO 6 9 12μM
PAI-1
PAI-1
45kd
45kd
GAPDH
GAPDH
35kd
35kd

## Slide 5
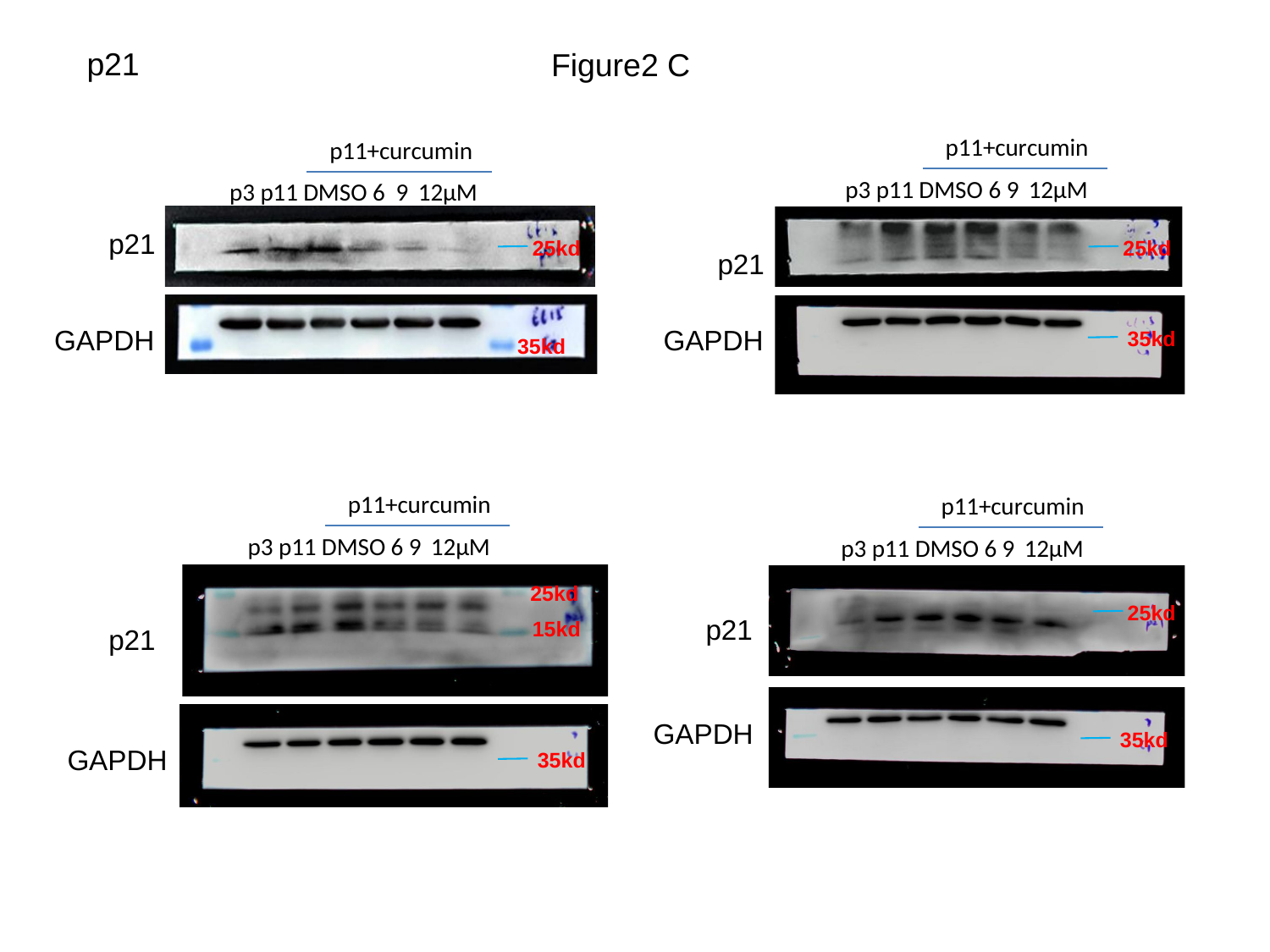

p21
Figure2 C
p11+curcumin
p11+curcumin
p3 p11 DMSO 6 9 12μM
p3 p11 DMSO 6 9 12μM
p21
25kd
25kd
p21
GAPDH
GAPDH
35kd
35kd
p11+curcumin
p11+curcumin
p3 p11 DMSO 6 9 12μM
p3 p11 DMSO 6 9 12μM
25kd
25kd
p21
15kd
p21
GAPDH
35kd
GAPDH
35kd

## Slide 6
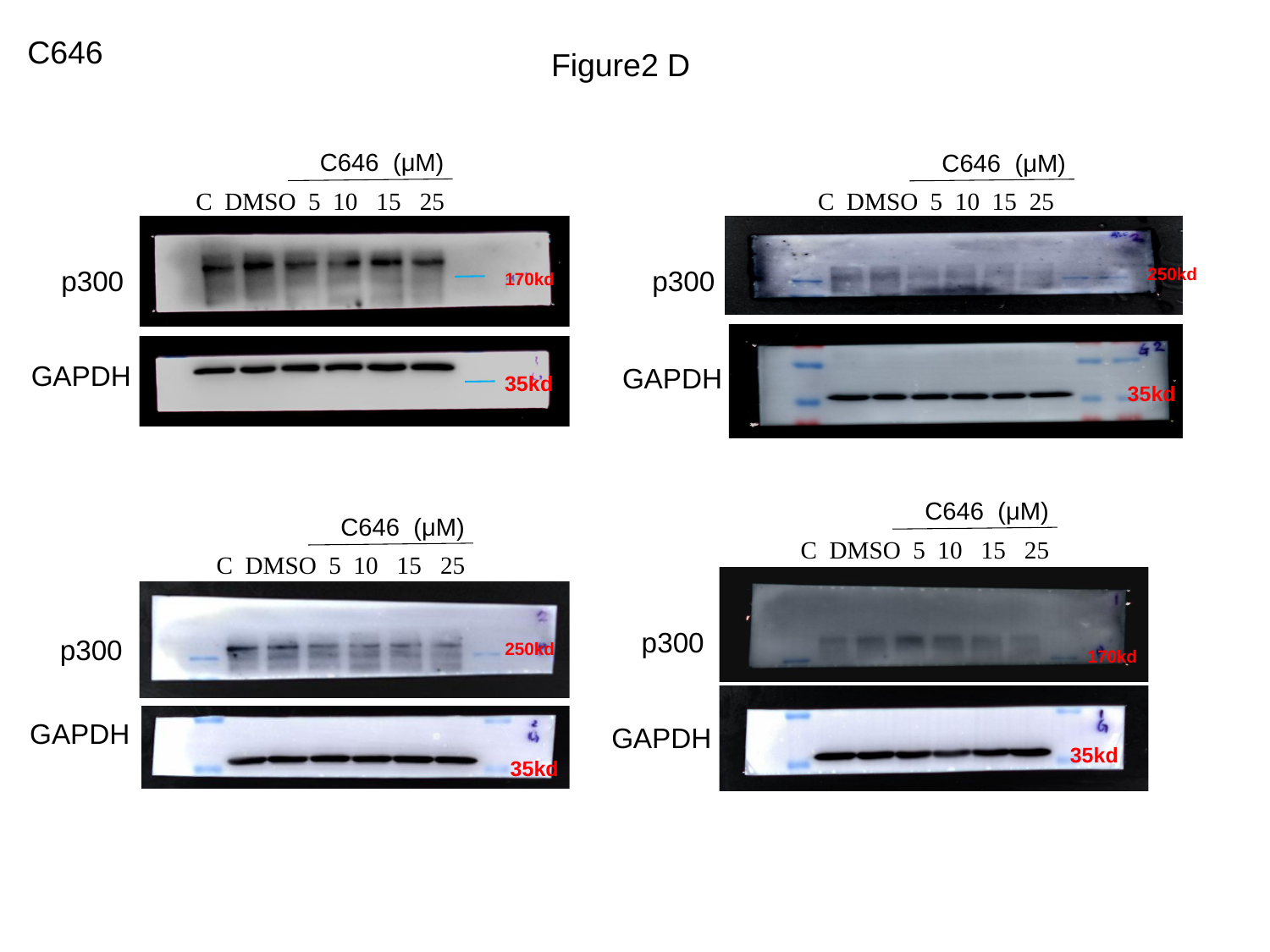

C646
Figure2 D
C646 (μM)
C646 (μM)
 C DMSO 5 10 15 25
 C DMSO 5 10 15 25
250kd
p300
p300
170kd
GAPDH
GAPDH
35kd
35kd
C646 (μM)
C646 (μM)
 C DMSO 5 10 15 25
 C DMSO 5 10 15 25
p300
p300
250kd
170kd
GAPDH
GAPDH
35kd
35kd

## Slide 7
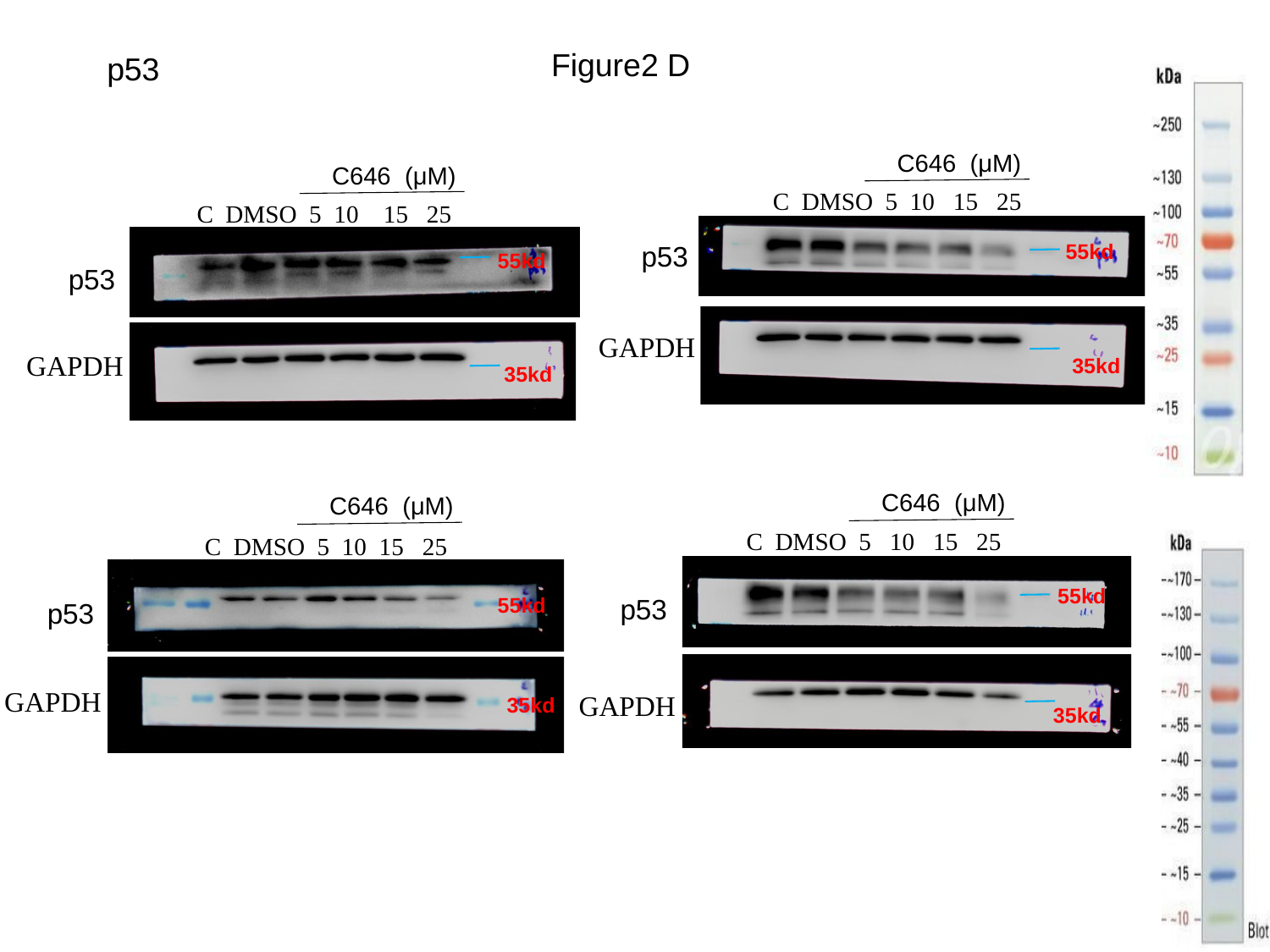

Figure2 D
p53
C646 (μM)
C646 (μM)
 C DMSO 5 10 15 25
 C DMSO 5 10 15 25
55kd
p53
55kd
p53
GAPDH
GAPDH
35kd
35kd
C646 (μM)
C646 (μM)
 C DMSO 5 10 15 25
 C DMSO 5 10 15 25
55kd
p53
55kd
p53
GAPDH
GAPDH
35kd
35kd

## Slide 8
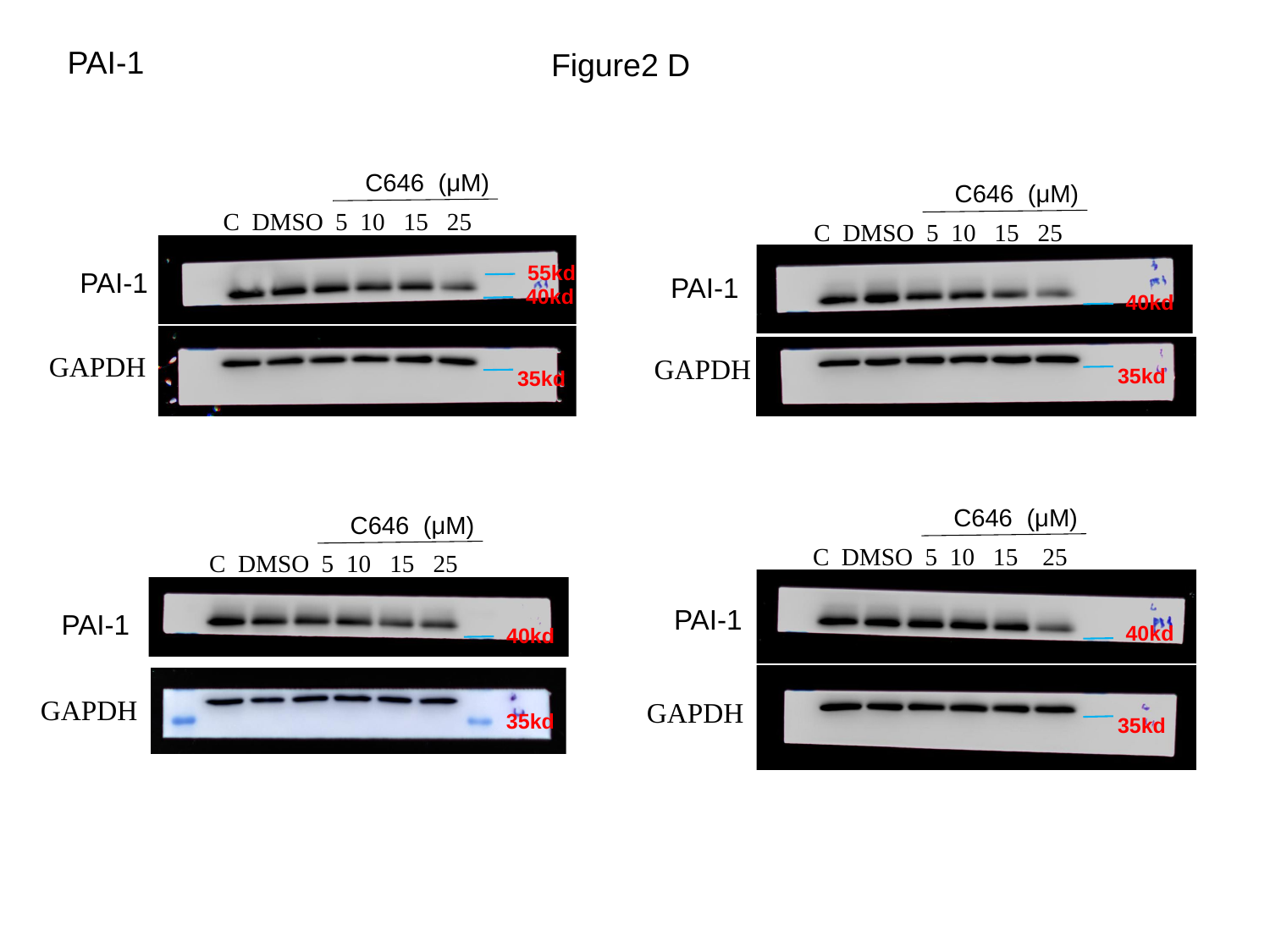

PAI-1
Figure2 D
C646 (μM)
C646 (μM)
 C DMSO 5 10 15 25
 C DMSO 5 10 15 25
55kd
PAI-1
PAI-1
40kd
40kd
GAPDH
GAPDH
35kd
35kd
C646 (μM)
C646 (μM)
 C DMSO 5 10 15 25
 C DMSO 5 10 15 25
PAI-1
PAI-1
40kd
40kd
GAPDH
GAPDH
35kd
35kd

## Slide 9
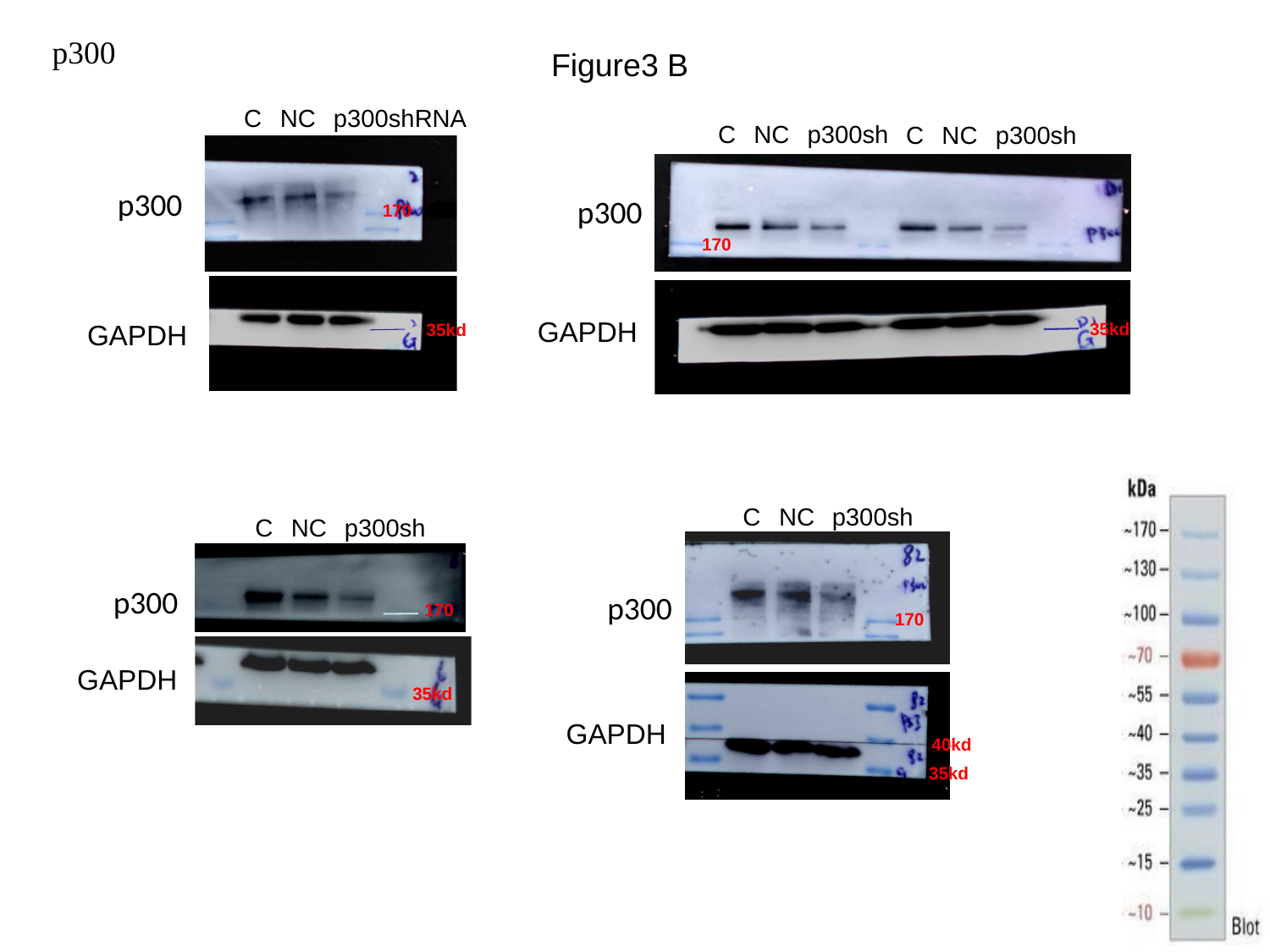

p300
Figure3 B
C
NC
p300shRNA
C
NC
p300sh
C
NC
p300sh
p300
p300
170
170
GAPDH
35kd
GAPDH
35kd
C
NC
p300sh
C
NC
p300sh
p300
p300
170
170
GAPDH
35kd
GAPDH
40kd
35kd

## Slide 10
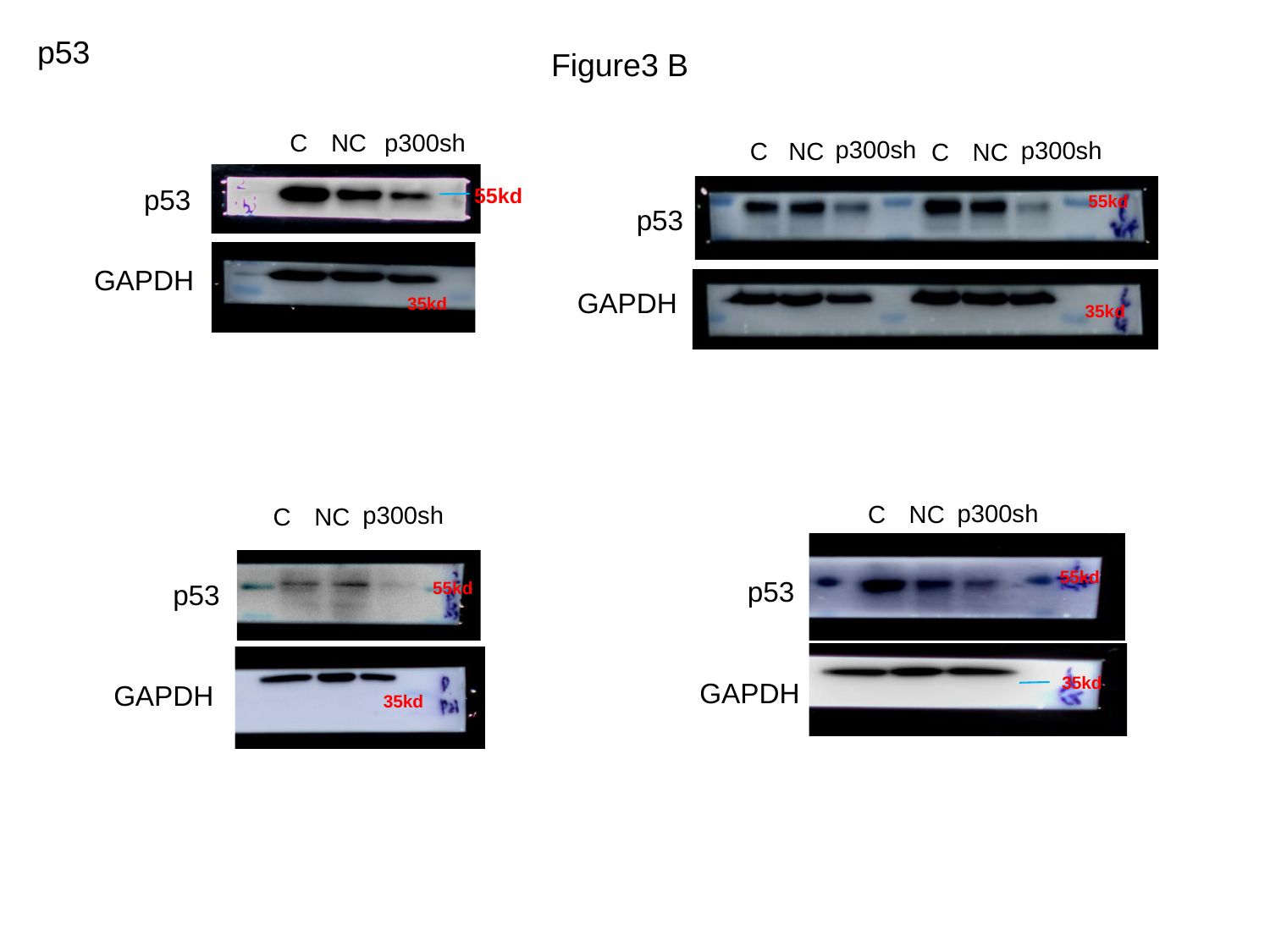

p53
Figure3 B
C
NC
p300sh
p300sh
p300sh
C
NC
C
NC
p53
55kd
55kd
p53
GAPDH
GAPDH
35kd
35kd
p300sh
C
NC
p300sh
C
NC
55kd
p53
p53
55kd
35kd
GAPDH
GAPDH
35kd

## Slide 11
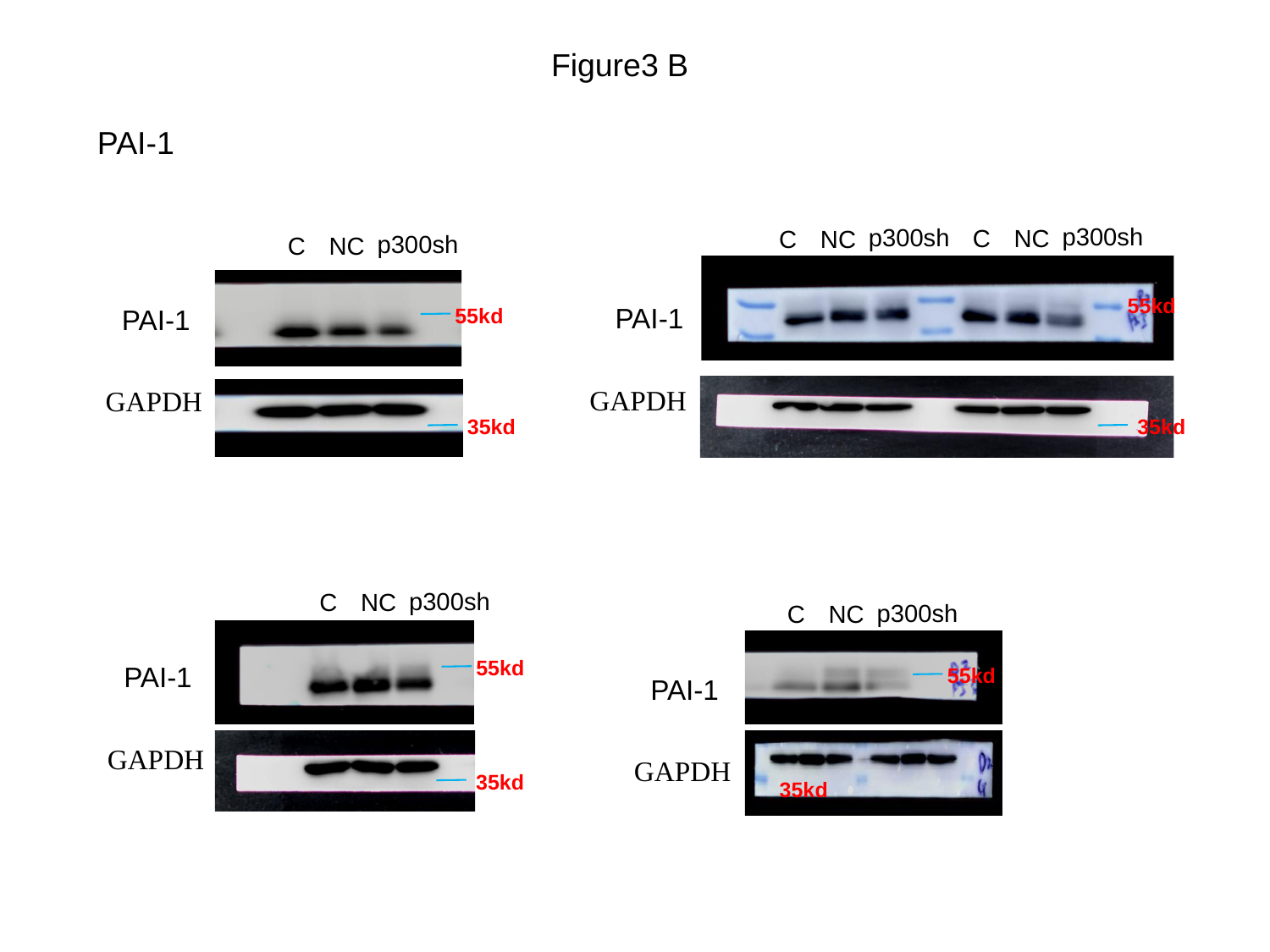

Figure3 B
PAI-1
p300sh
p300sh
C
NC
C
NC
p300sh
C
NC
55kd
PAI-1
PAI-1
55kd
GAPDH
GAPDH
35kd
35kd
p300sh
C
NC
p300sh
C
NC
55kd
PAI-1
55kd
PAI-1
GAPDH
GAPDH
35kd
35kd

## Slide 12
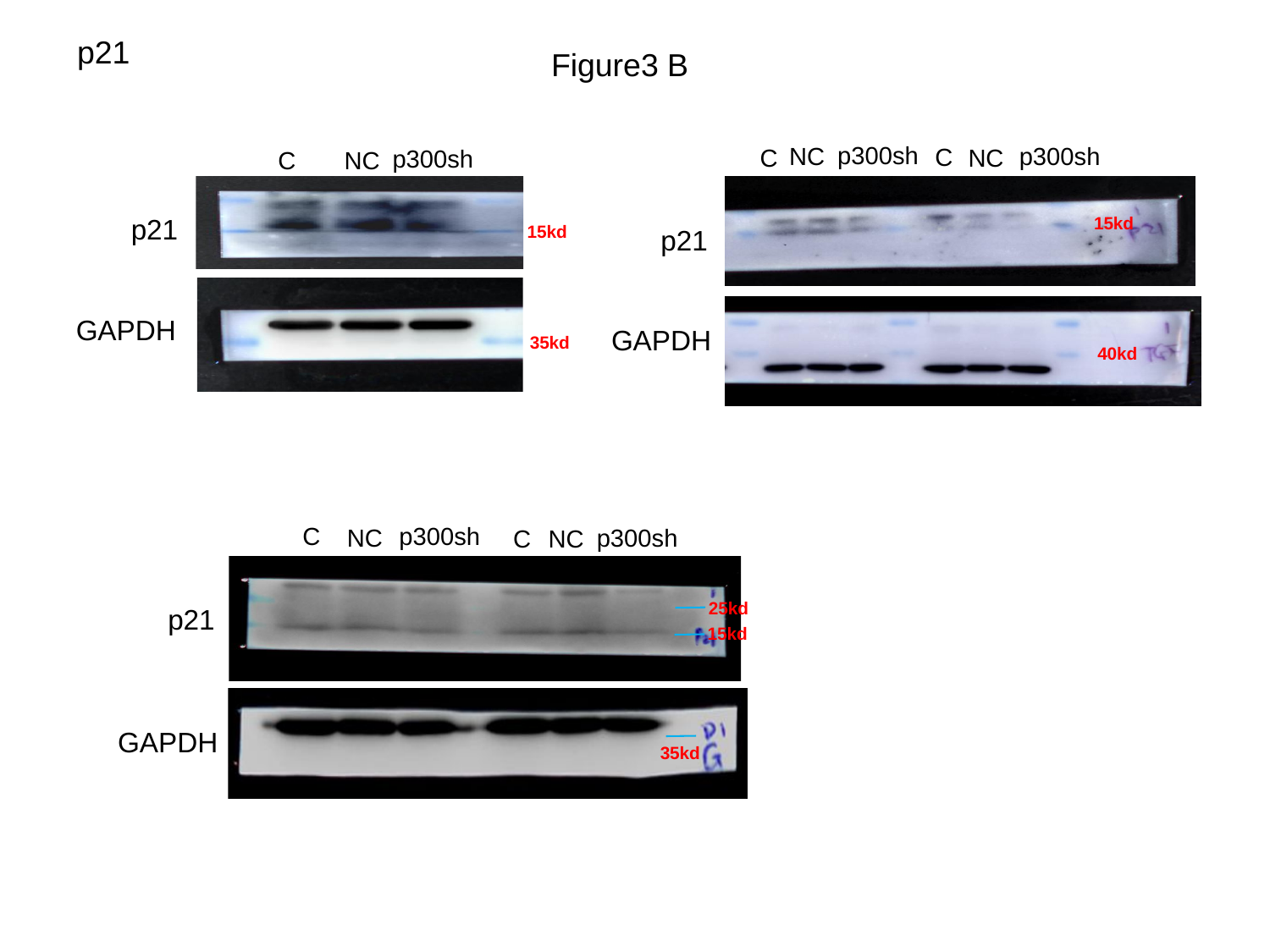

p21
Figure3 B
p300sh
NC
p300sh
C
C
NC
p300sh
C
NC
p21
15kd
15kd
p21
GAPDH
GAPDH
35kd
40kd
C
p300sh
NC
p300sh
C
NC
25kd
p21
15kd
GAPDH
35kd

## Slide 13
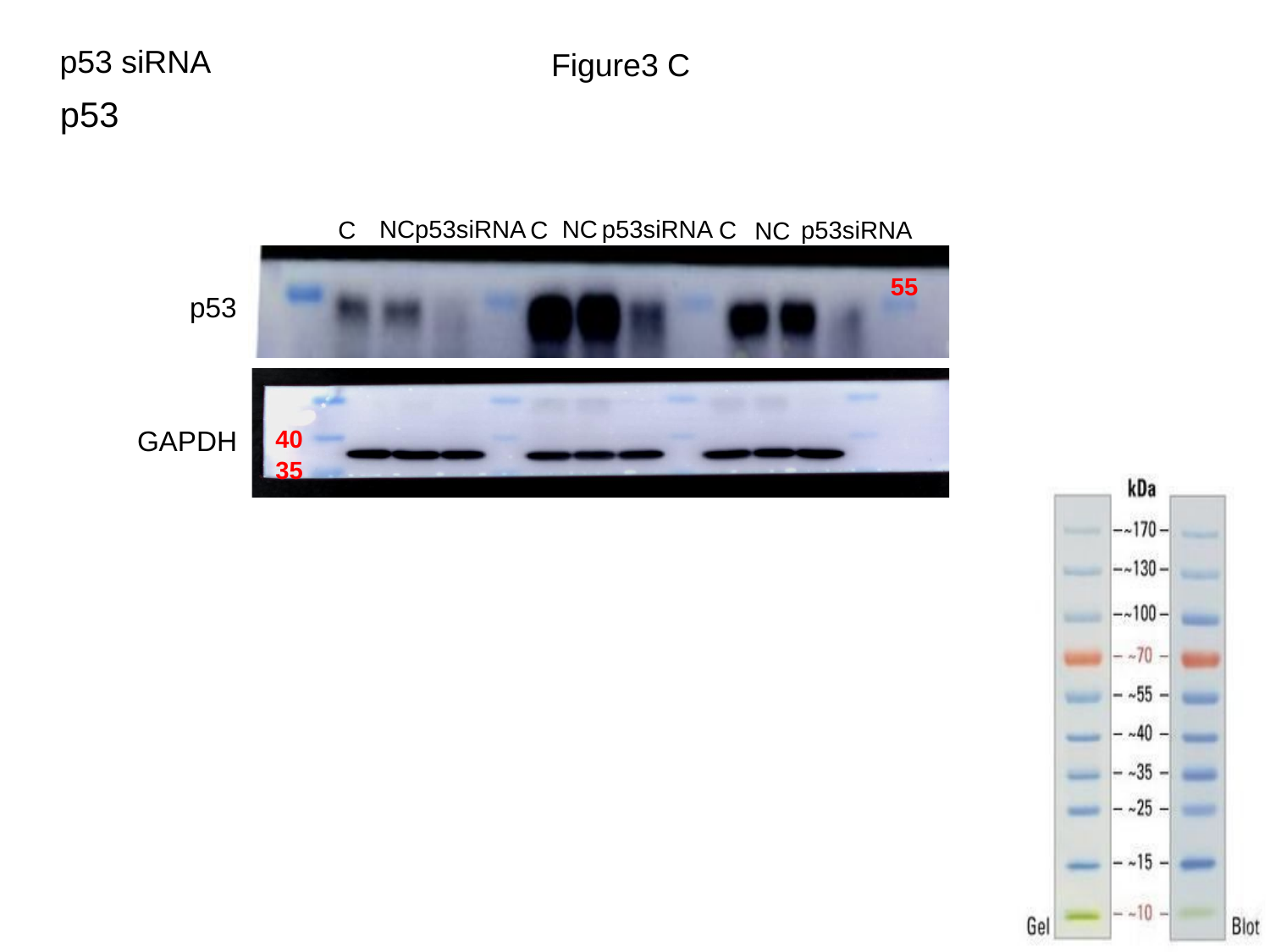

p53 siRNA
Figure3 C
p53
NC
p53siRNA
NC
p53siRNA
C
p53siRNA
C
C
NC
55
p53
GAPDH
40
35

## Slide 14
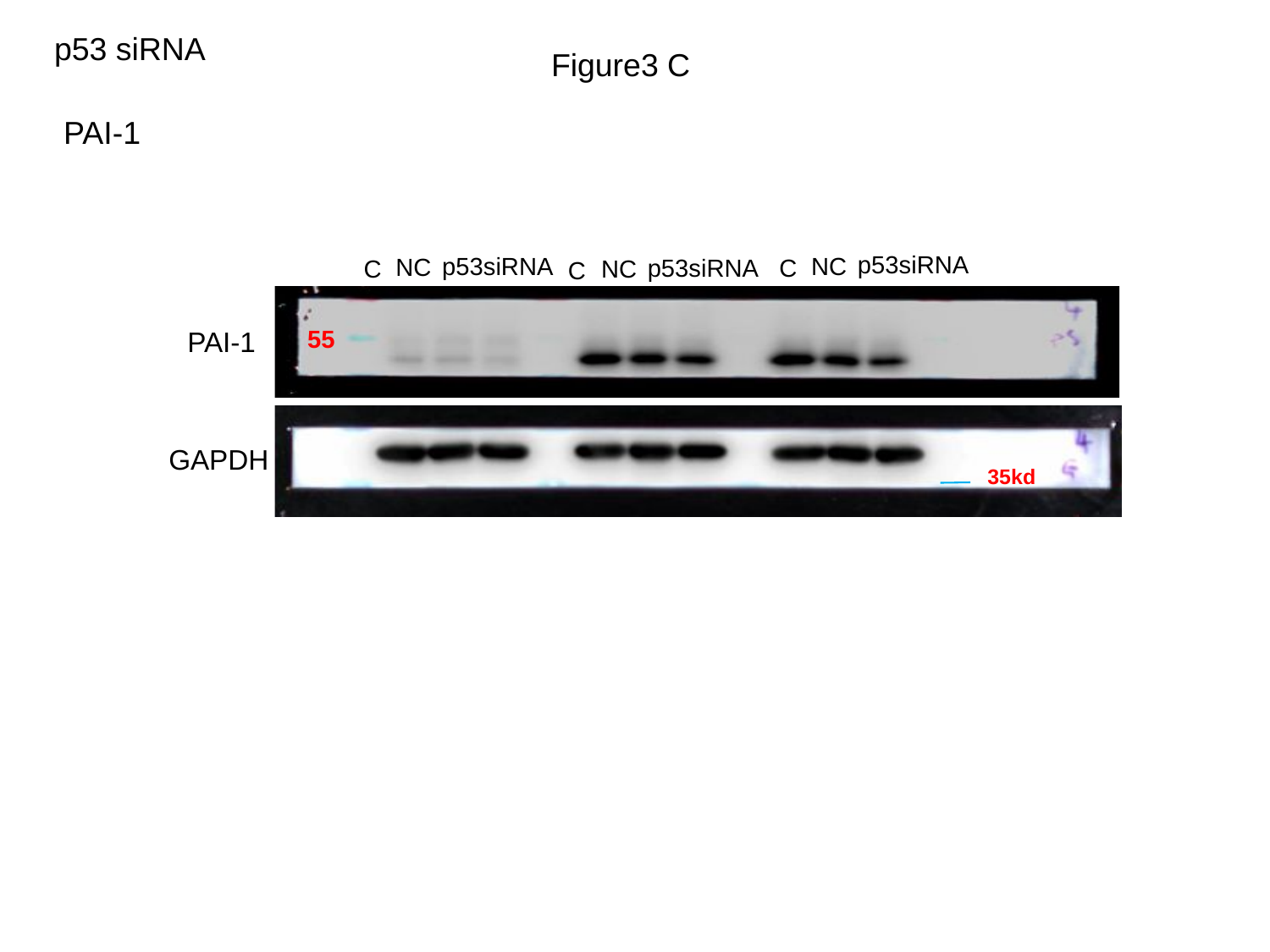

p53 siRNA
Figure3 C
PAI-1
p53siRNA
p53siRNA
NC
NC
C
p53siRNA
C
NC
C
55
PAI-1
GAPDH
35kd

## Slide 15
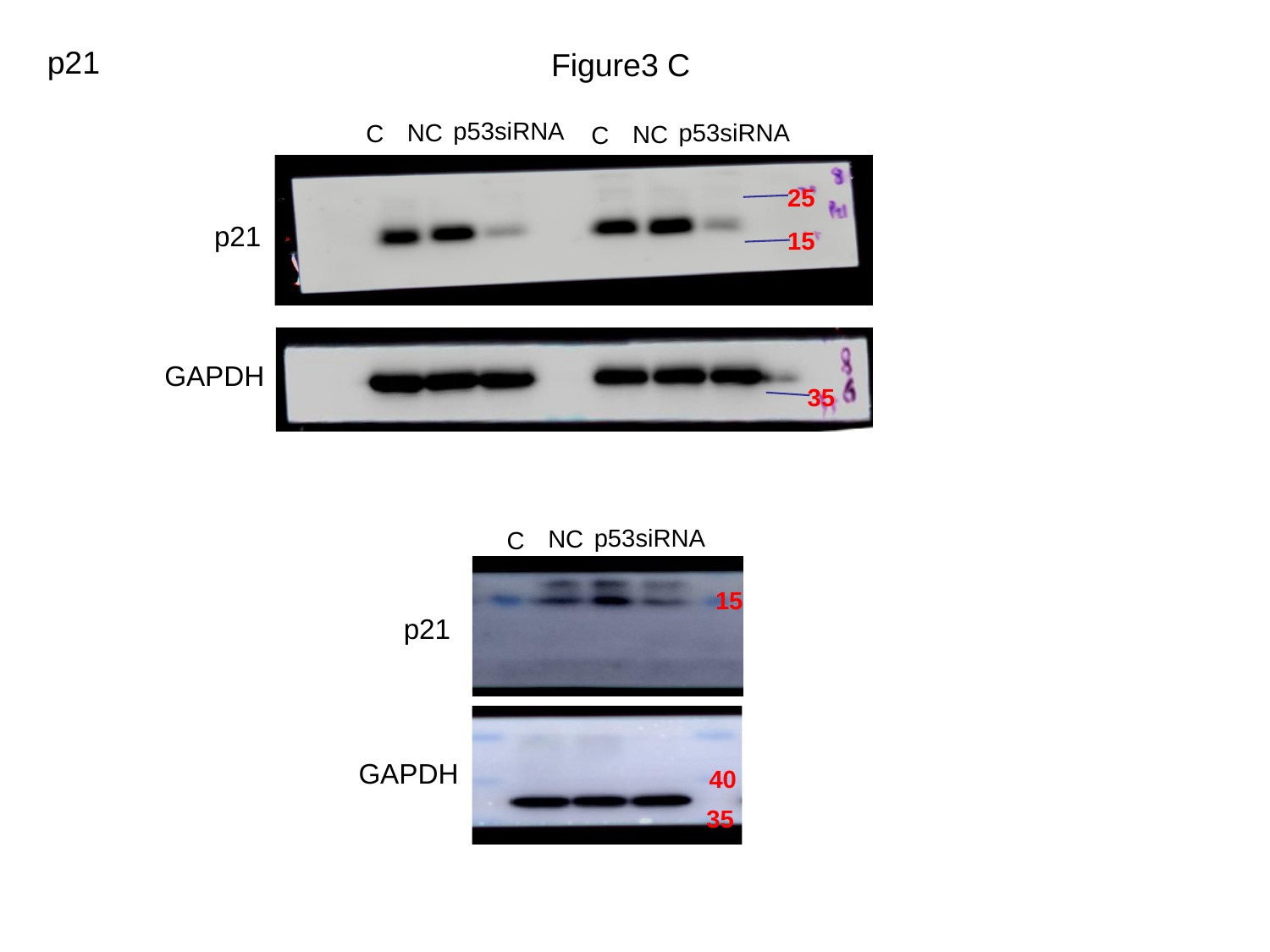

p21
Figure3 C
p53siRNA
NC
p53siRNA
C
NC
C
25
p21
15
GAPDH
35
p53siRNA
NC
C
15
p21
GAPDH
40
35

## Slide 16
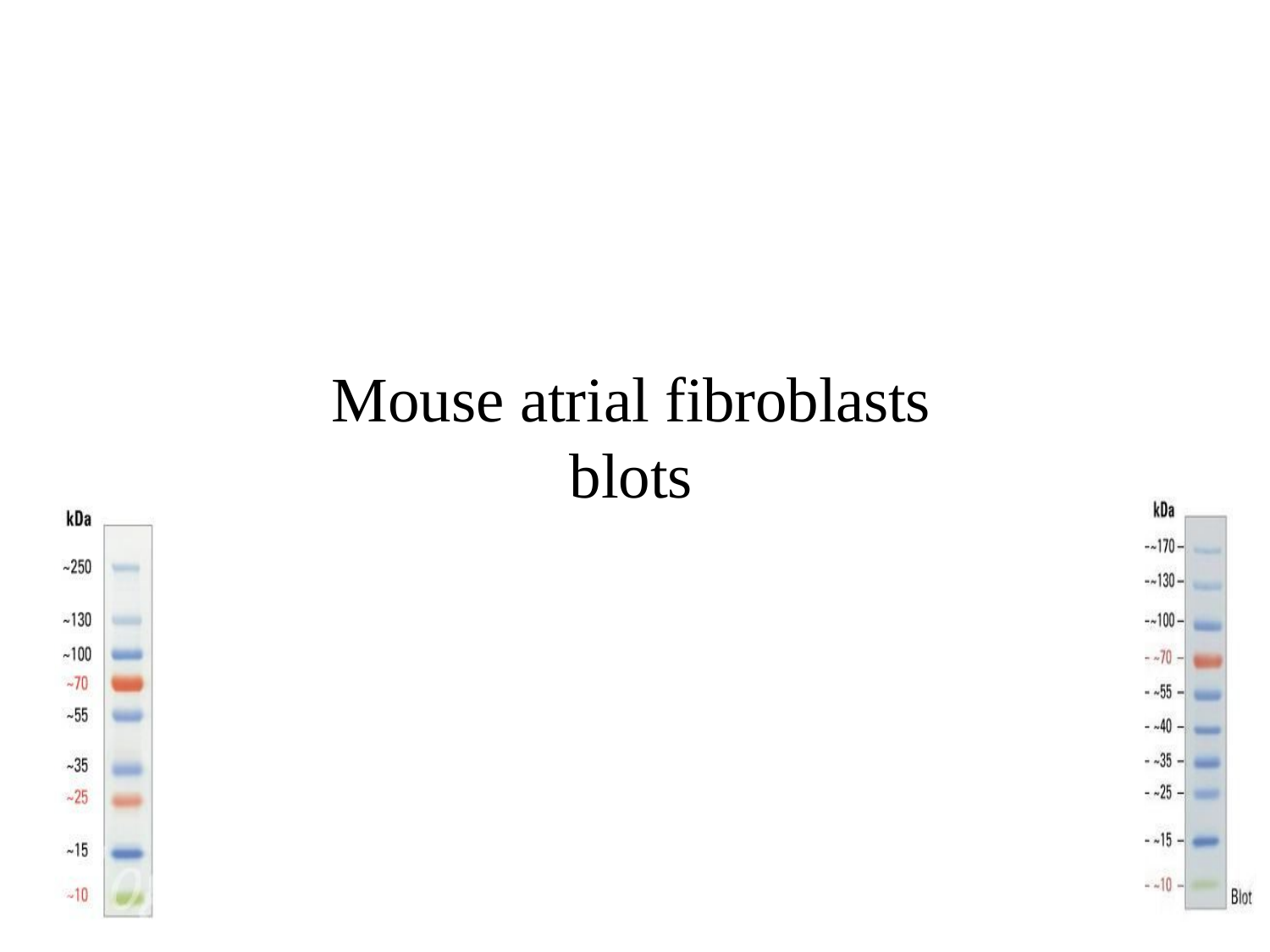

Mouse atrial fibroblasts blots

## Slide 17
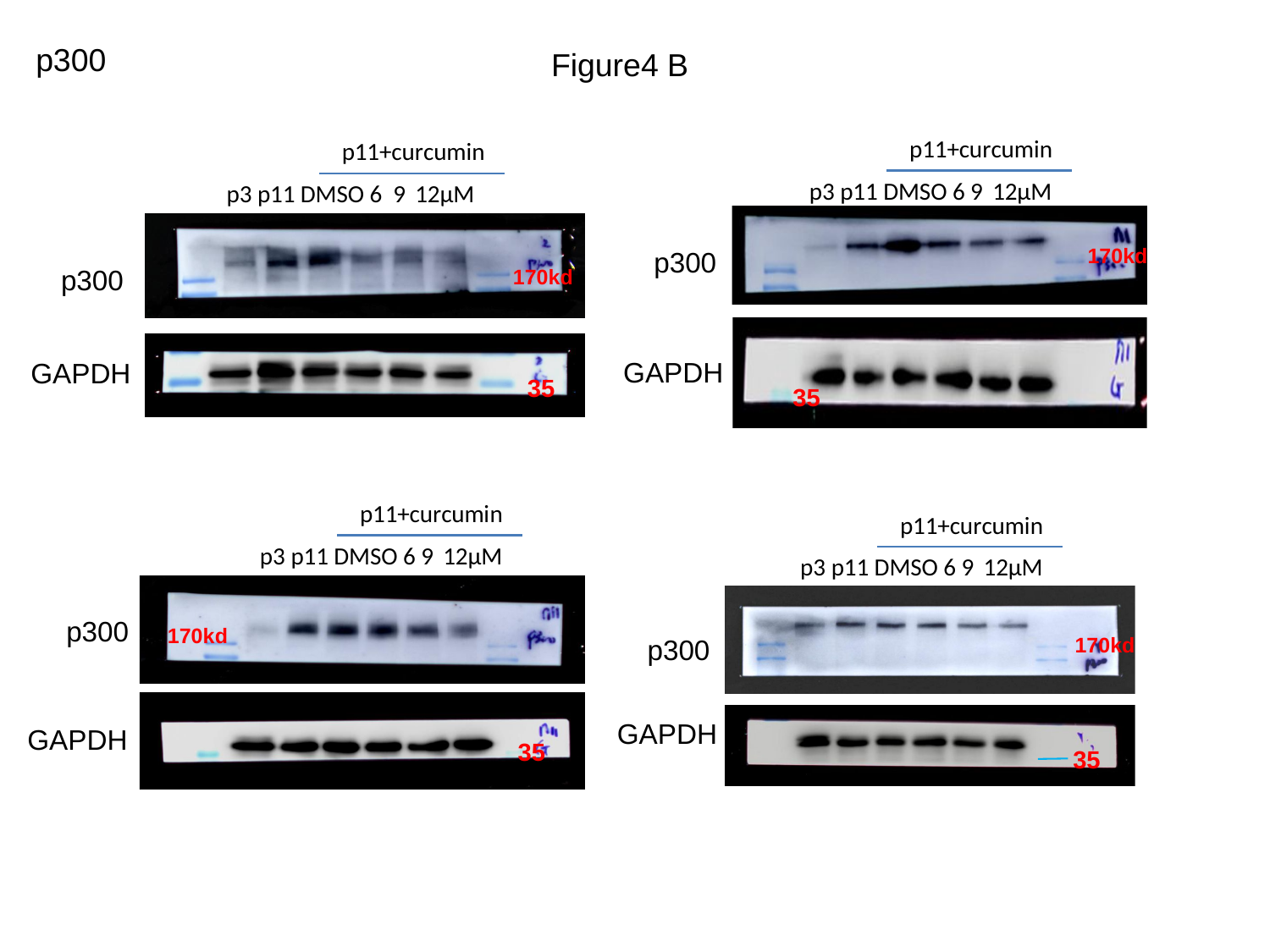

p300
Figure4 B
p11+curcumin
p11+curcumin
p3 p11 DMSO 6 9 12μM
p3 p11 DMSO 6 9 12μM
170kd
p300
p300
170kd
GAPDH
GAPDH
35
35
p11+curcumin
p11+curcumin
p3 p11 DMSO 6 9 12μM
p3 p11 DMSO 6 9 12μM
p300
170kd
p300
170kd
GAPDH
GAPDH
35
35

## Slide 18
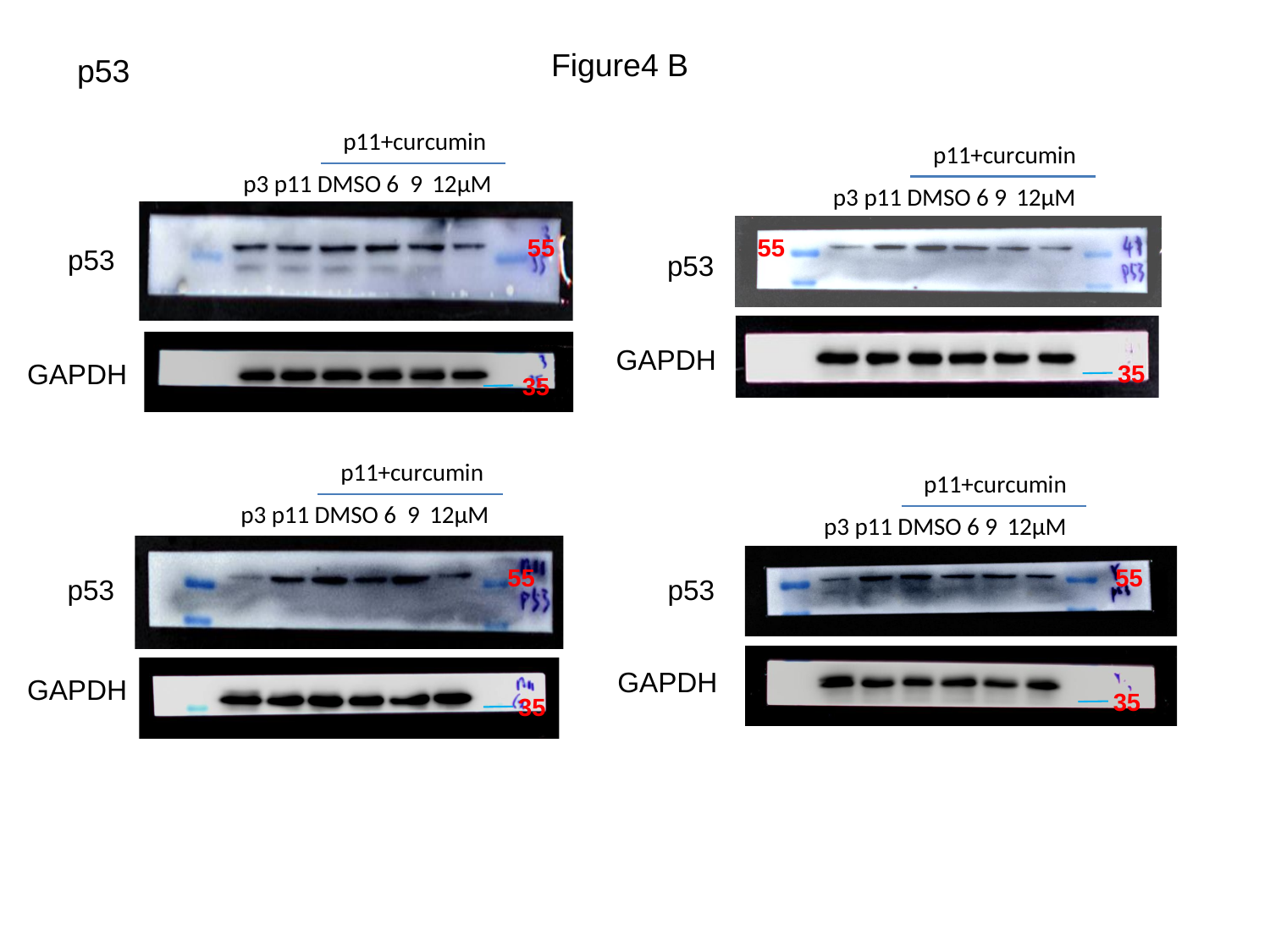

Figure4 B
p53
p11+curcumin
p11+curcumin
p3 p11 DMSO 6 9 12μM
p3 p11 DMSO 6 9 12μM
55
55
p53
p53
GAPDH
GAPDH
35
35
p11+curcumin
p11+curcumin
p3 p11 DMSO 6 9 12μM
p3 p11 DMSO 6 9 12μM
55
55
p53
p53
GAPDH
GAPDH
35
35

## Slide 19
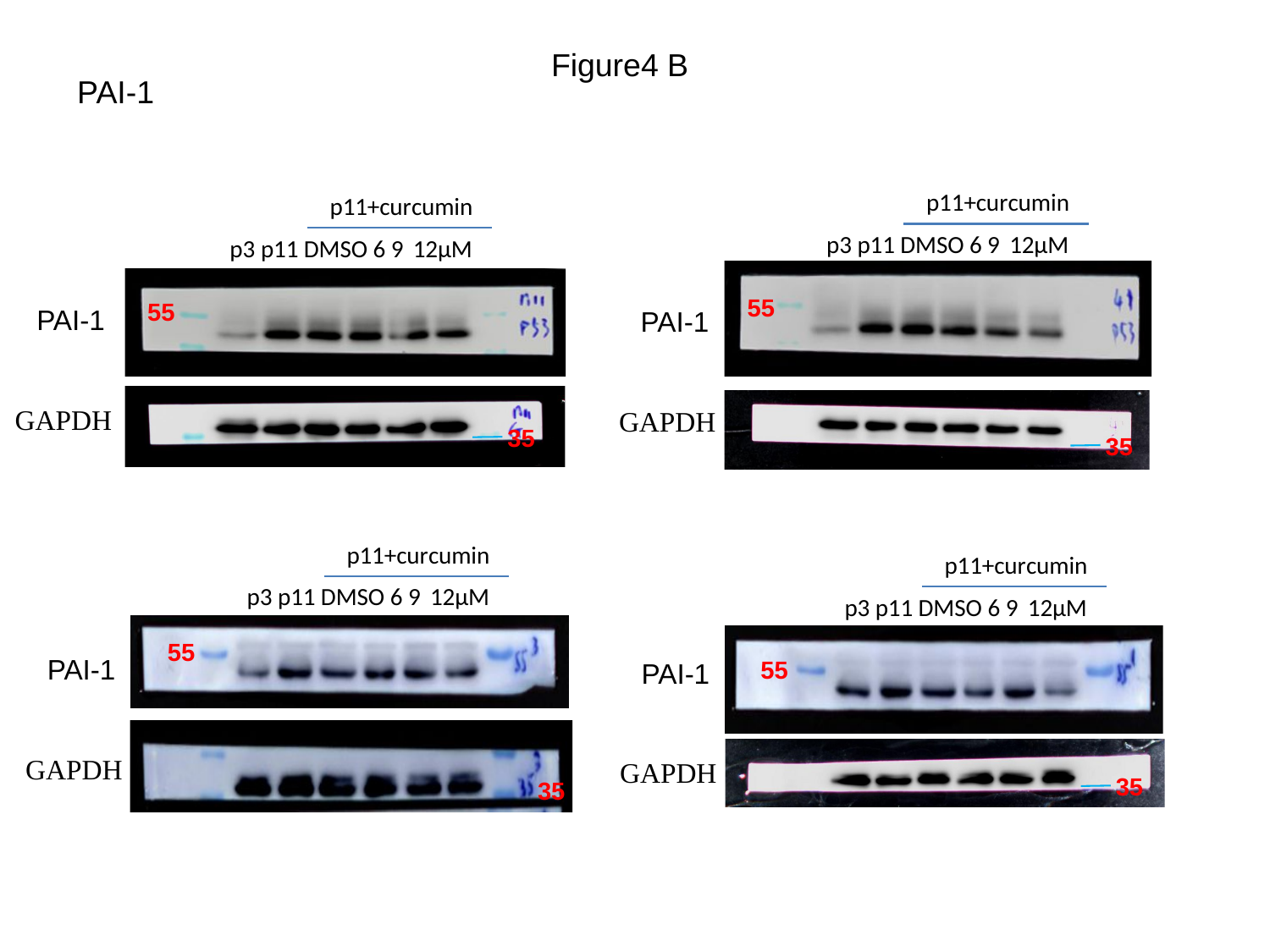

Figure4 B
PAI-1
p11+curcumin
p11+curcumin
p3 p11 DMSO 6 9 12μM
p3 p11 DMSO 6 9 12μM
55
55
PAI-1
PAI-1
GAPDH
GAPDH
35
35
p11+curcumin
p11+curcumin
p3 p11 DMSO 6 9 12μM
p3 p11 DMSO 6 9 12μM
55
PAI-1
55
PAI-1
GAPDH
GAPDH
35
35

## Slide 20
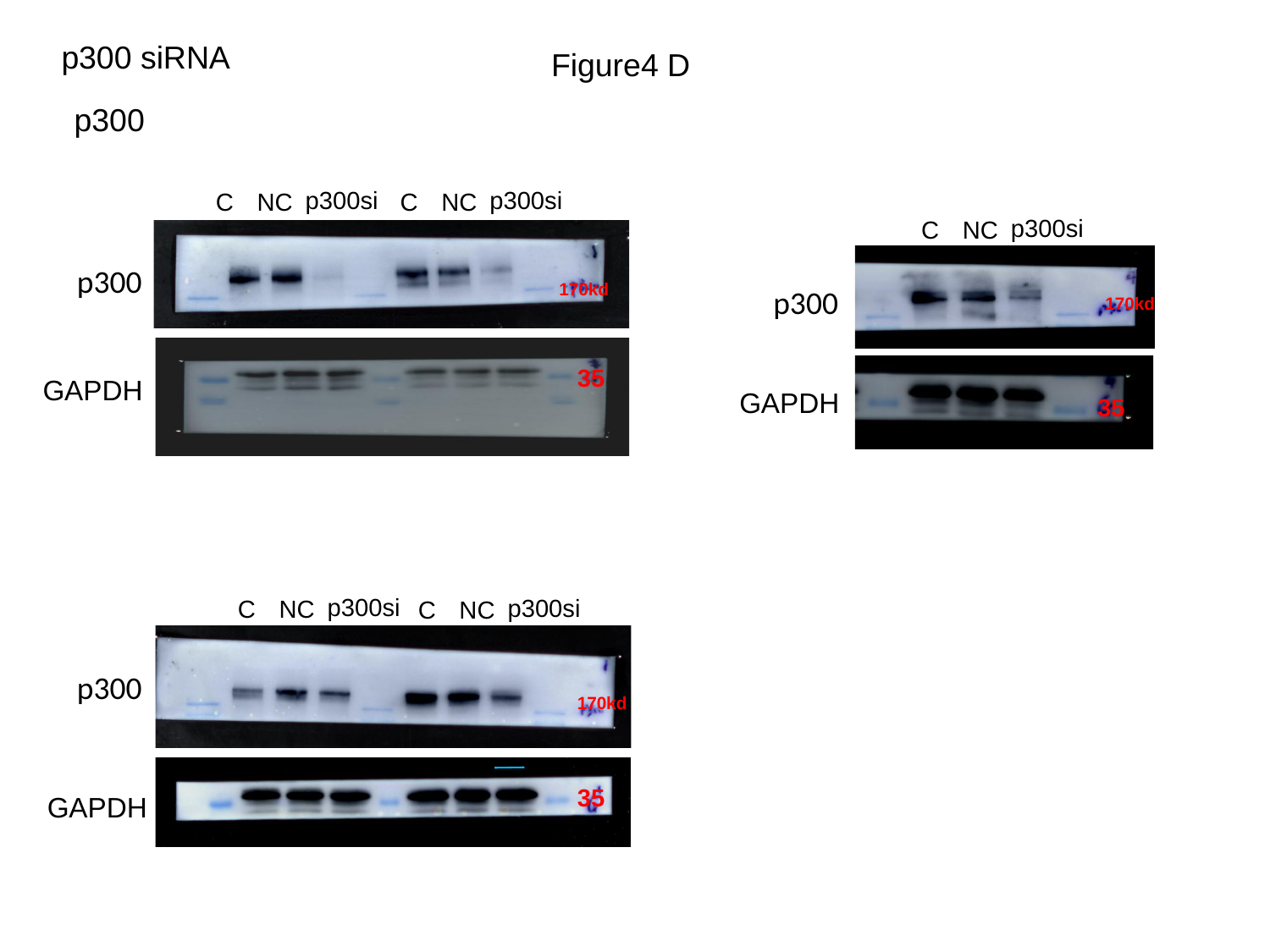

p300 siRNA
Figure4 D
p300
p300si
p300si
C
NC
C
NC
p300si
C
NC
p300
170kd
p300
170kd
35
GAPDH
GAPDH
35
p300si
p300si
C
NC
C
NC
p300
170kd
35
GAPDH

## Slide 21
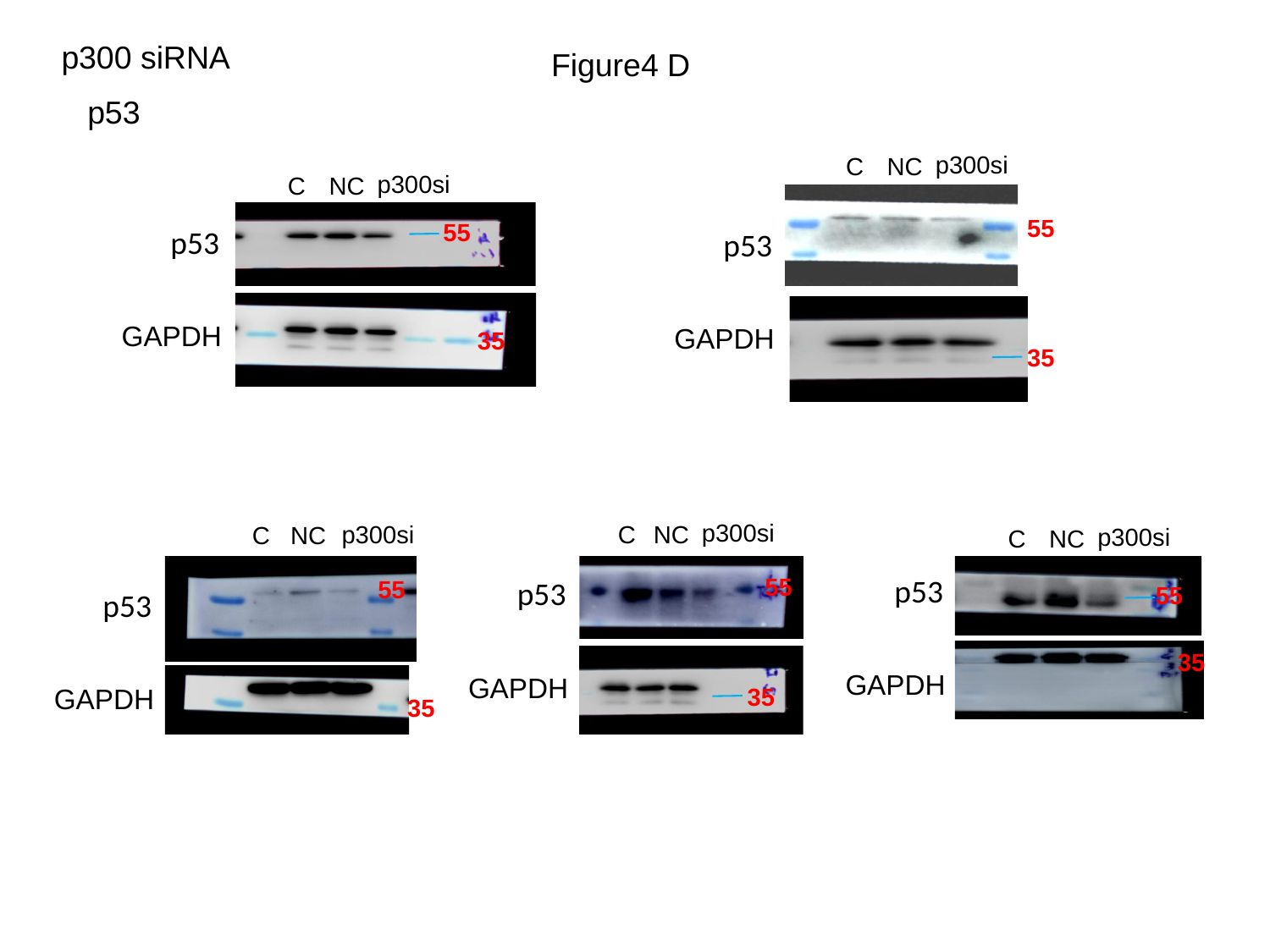

p300 siRNA
Figure4 D
p53
p300si
C
NC
p300si
C
NC
55
55
p53
p53
GAPDH
GAPDH
35
35
p300si
p300si
C
NC
C
NC
p300si
C
NC
55
p53
55
p53
55
p53
35
GAPDH
GAPDH
GAPDH
35
35

## Slide 22
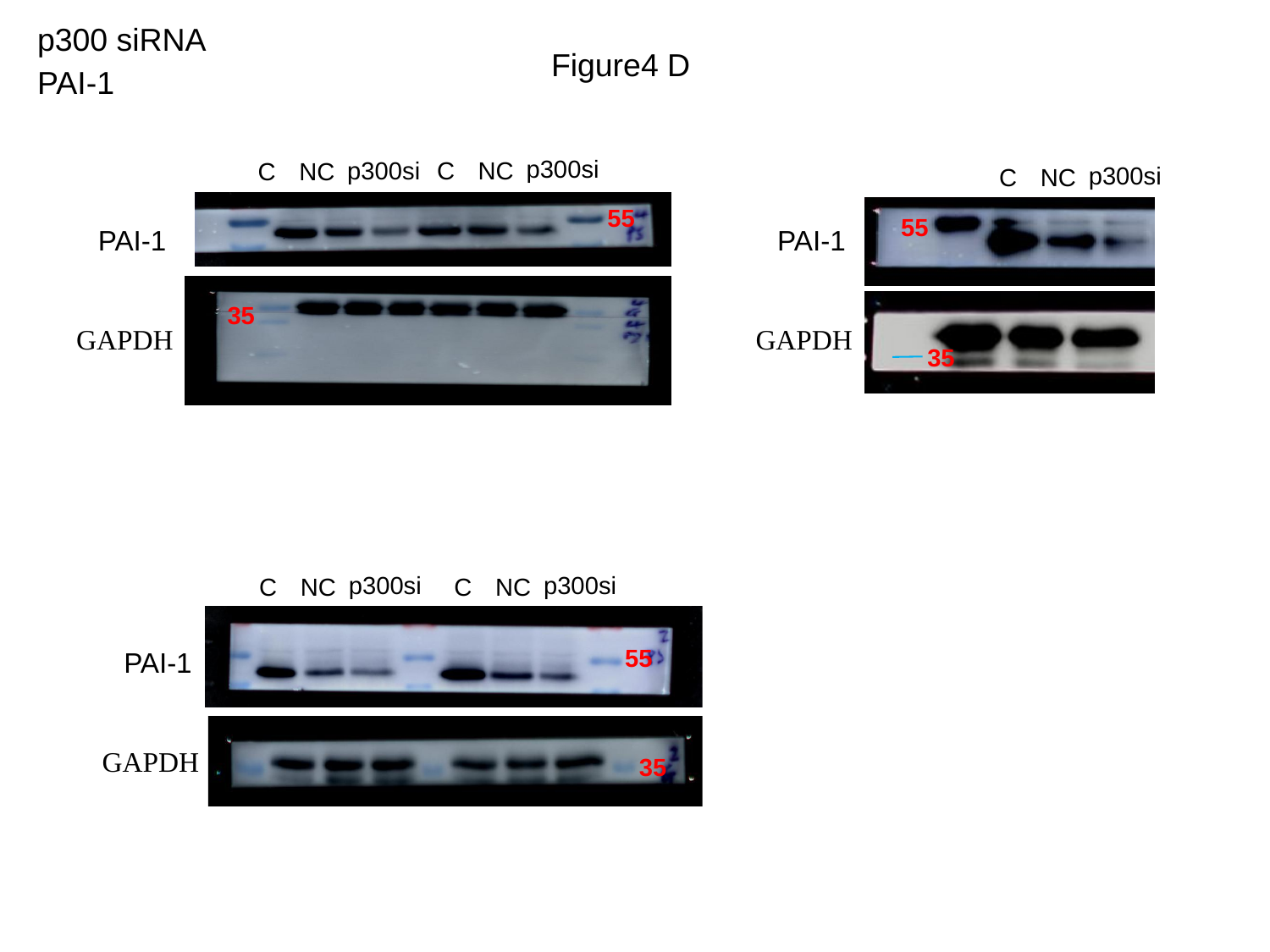

p300 siRNA
Figure4 D
PAI-1
p300si
p300si
C
NC
C
NC
p300si
C
NC
55
55
PAI-1
PAI-1
35
GAPDH
GAPDH
35
p300si
p300si
C
NC
C
NC
55
PAI-1
GAPDH
35

## Slide 23
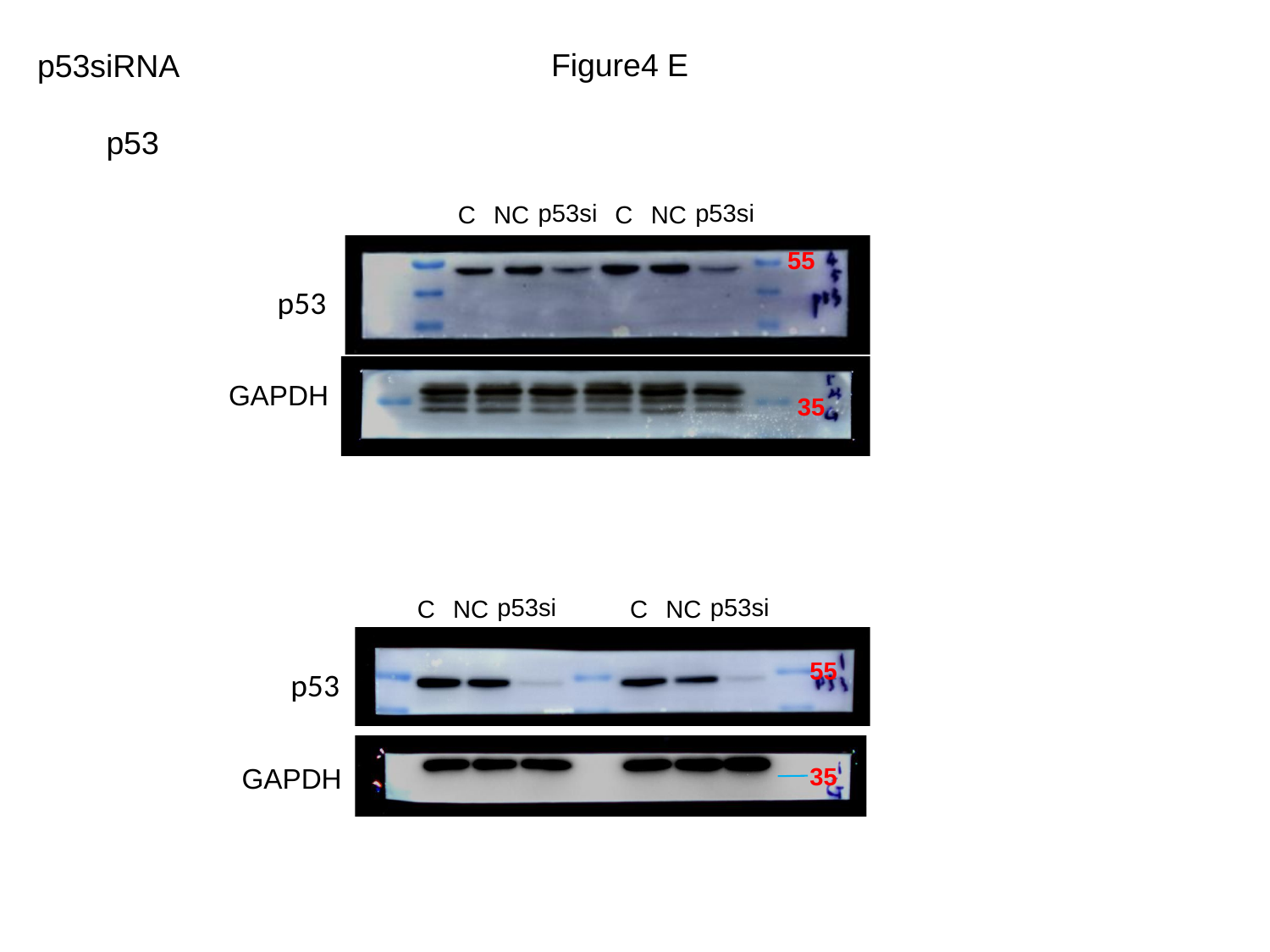

Figure4 E
p53siRNA
p53
p53si
p53si
C
NC
C
NC
55
p53
GAPDH
35
p53si
p53si
C
NC
C
NC
55
p53
35
GAPDH

## Slide 24
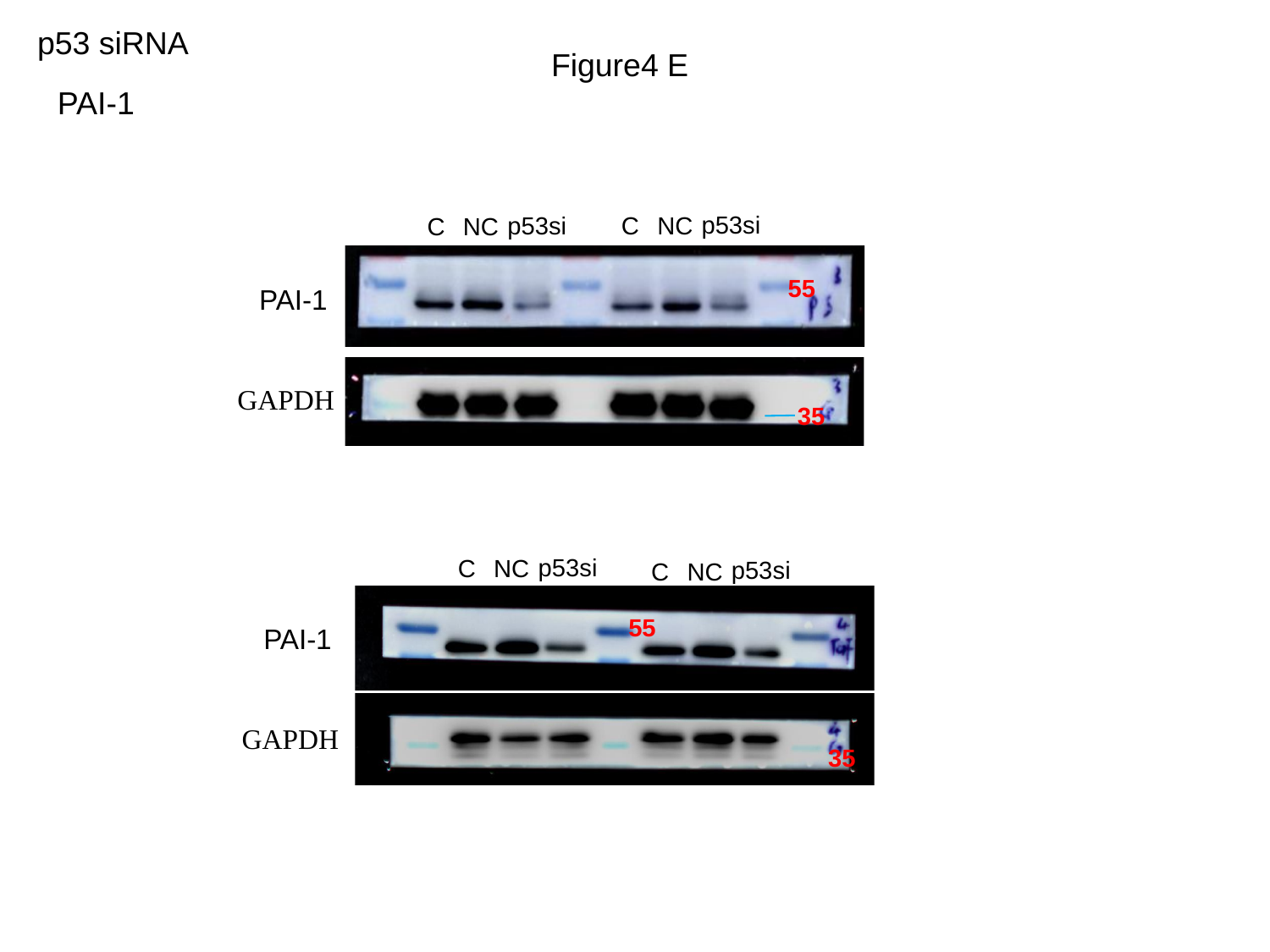

p53 siRNA
Figure4 E
PAI-1
p53si
p53si
C
NC
C
NC
55
PAI-1
GAPDH
35
p53si
C
NC
p53si
C
NC
55
PAI-1
GAPDH
35

## Slide 25
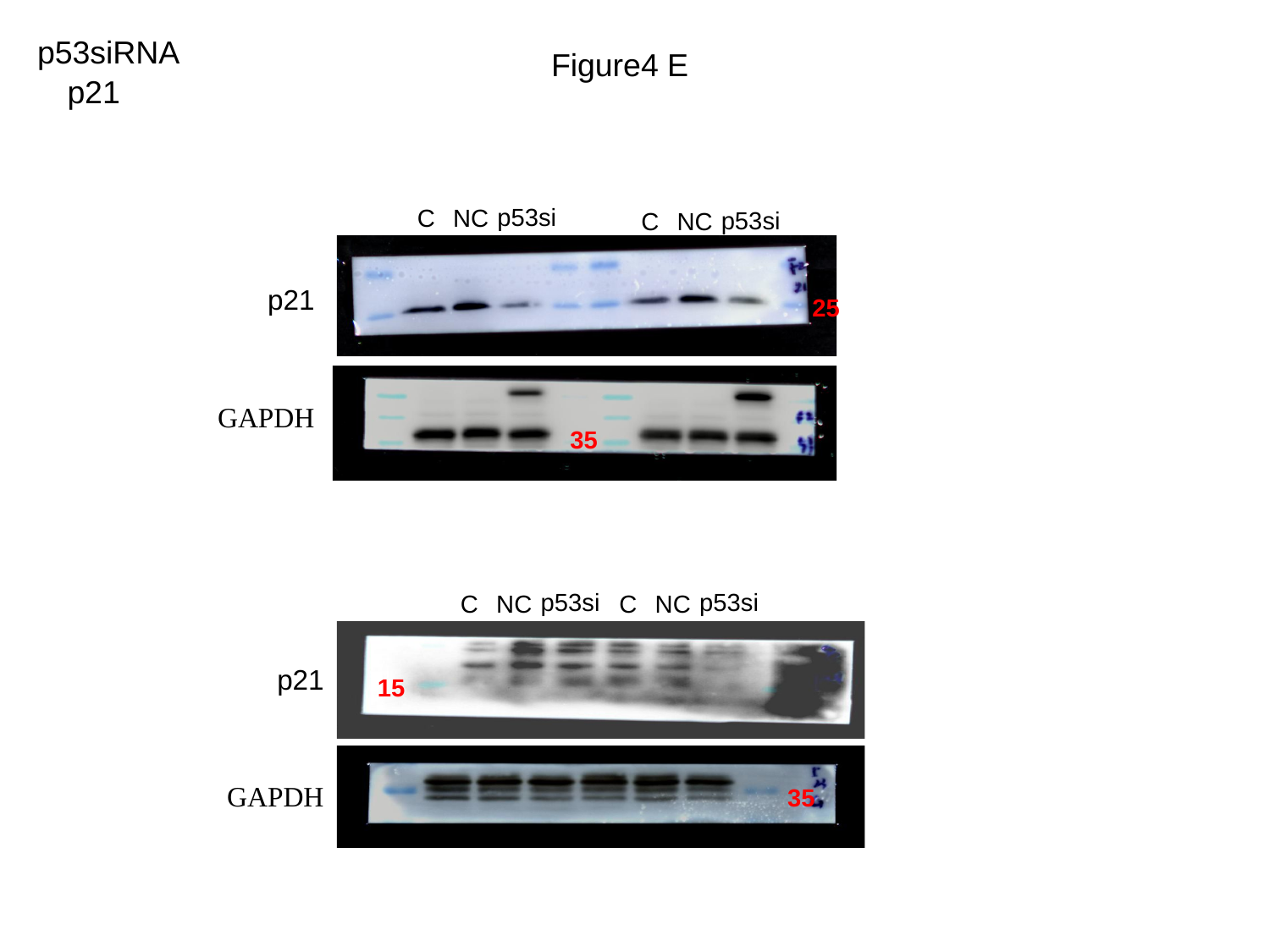

p53siRNA
Figure4 E
p21
p53si
C
NC
p53si
C
NC
p21
25
GAPDH
35
p53si
p53si
C
NC
C
NC
p21
15
GAPDH
35

## Slide 26
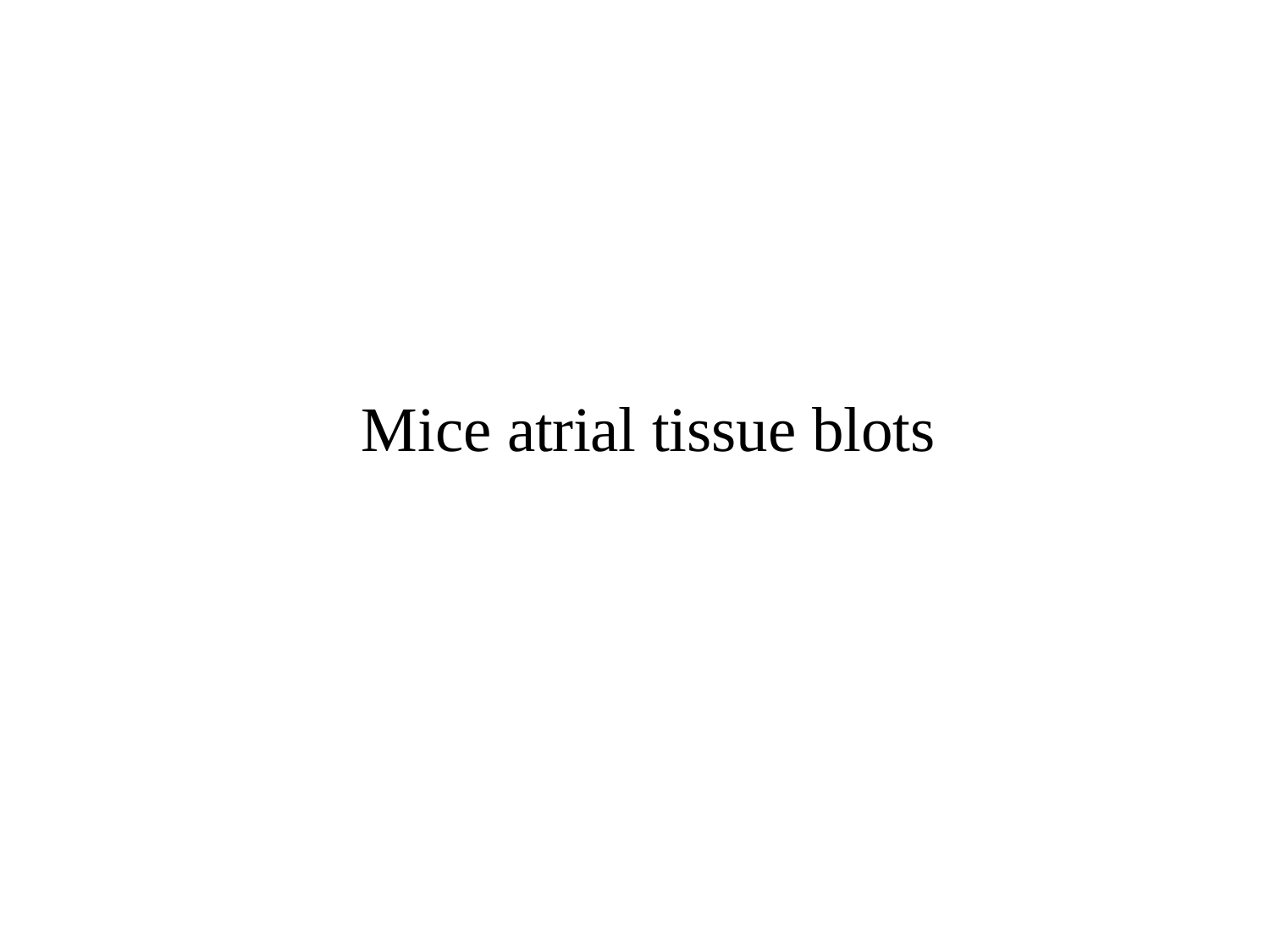

Mice atrial tissue blots

## Slide 27
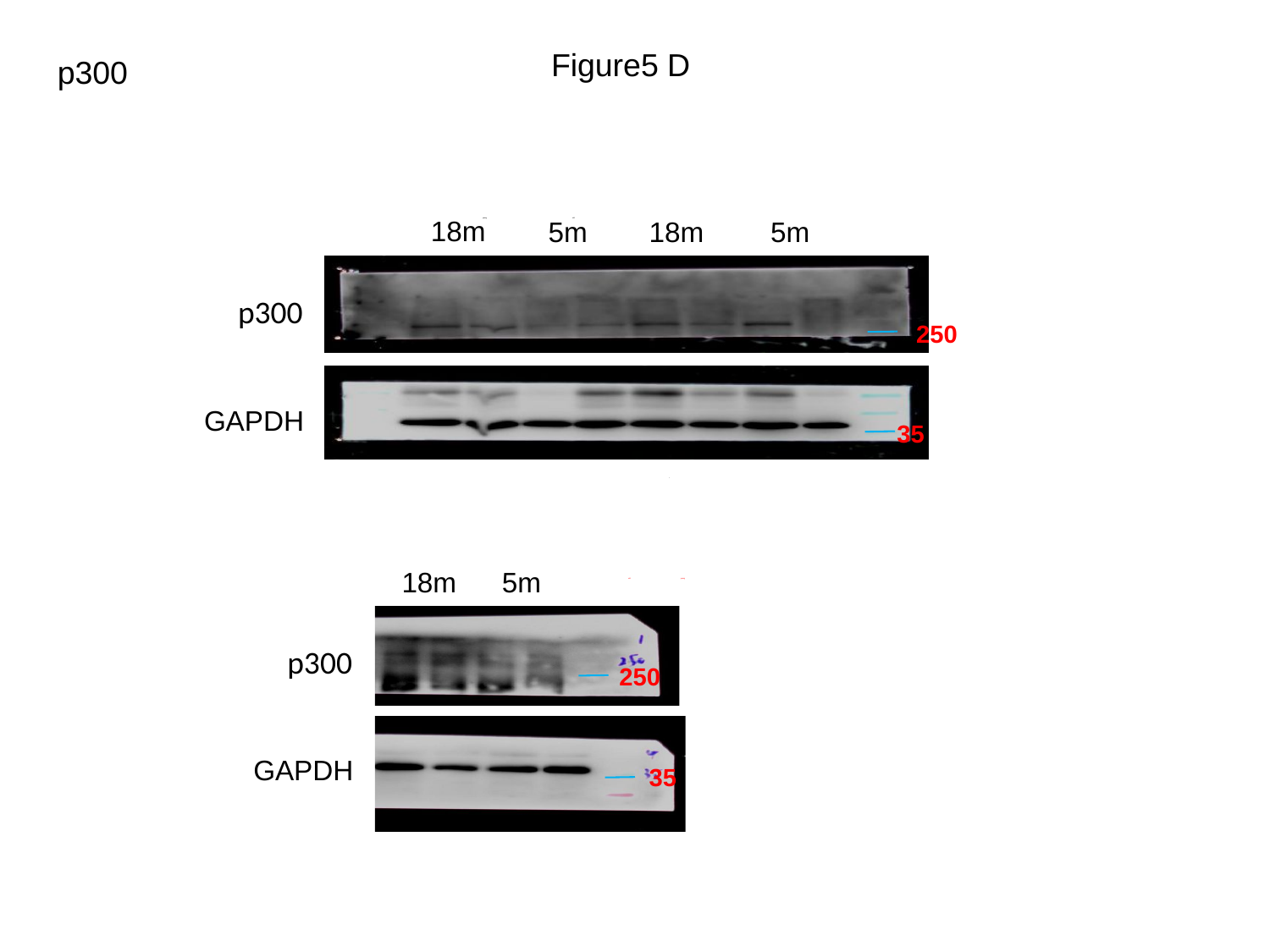

Figure5 D
p300
18m
5m
18m
5m
young
old
p300
250
GAPDH
35
18m
5m
old
young
p300
250
GAPDH
35

## Slide 28
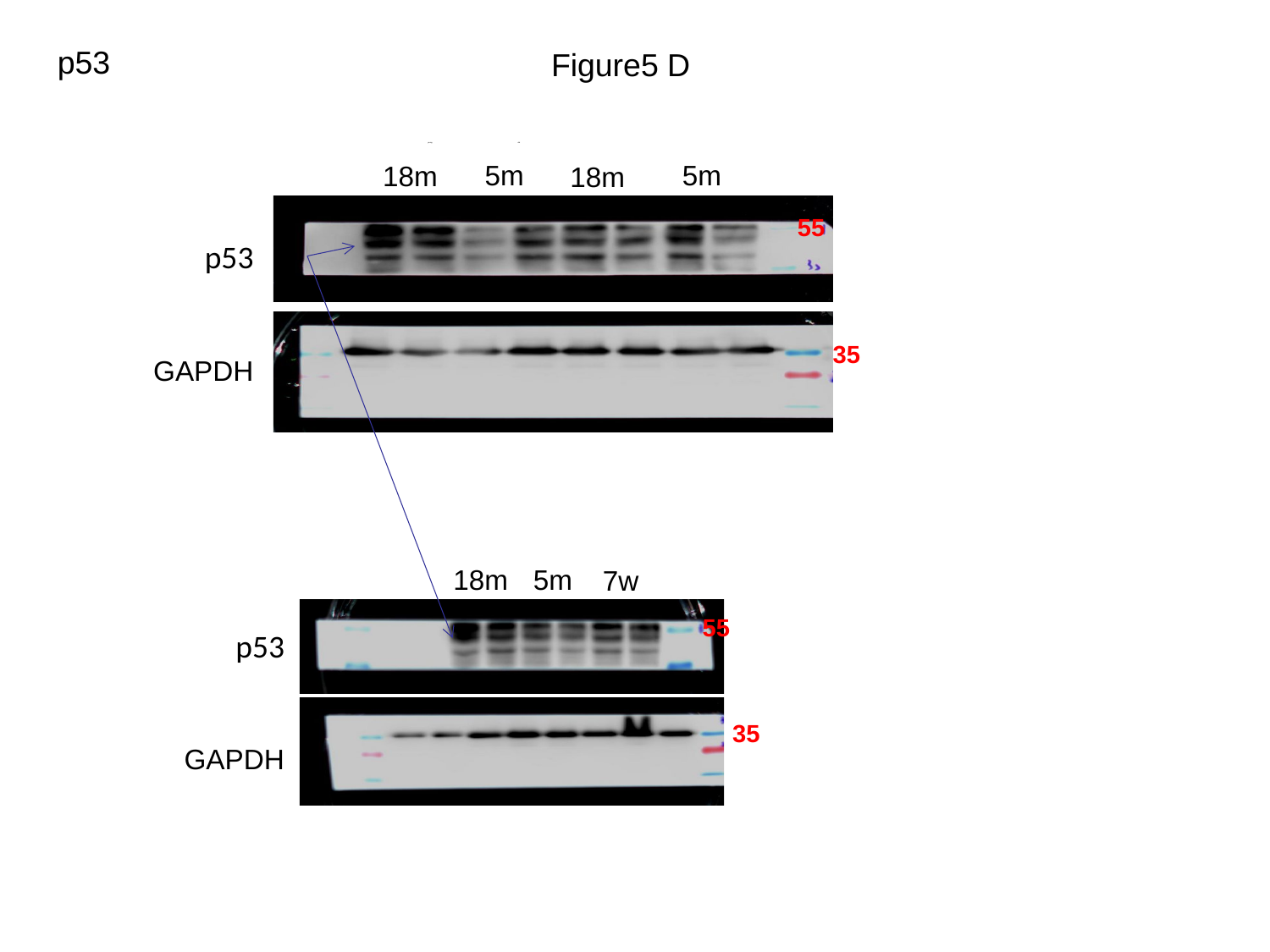

p53
Figure5 D
young
old
5m
5m
18m
18m
55
p53
p53
35
GAPDH
18m
5m
7w
55
p53
35
GAPDH

## Slide 29
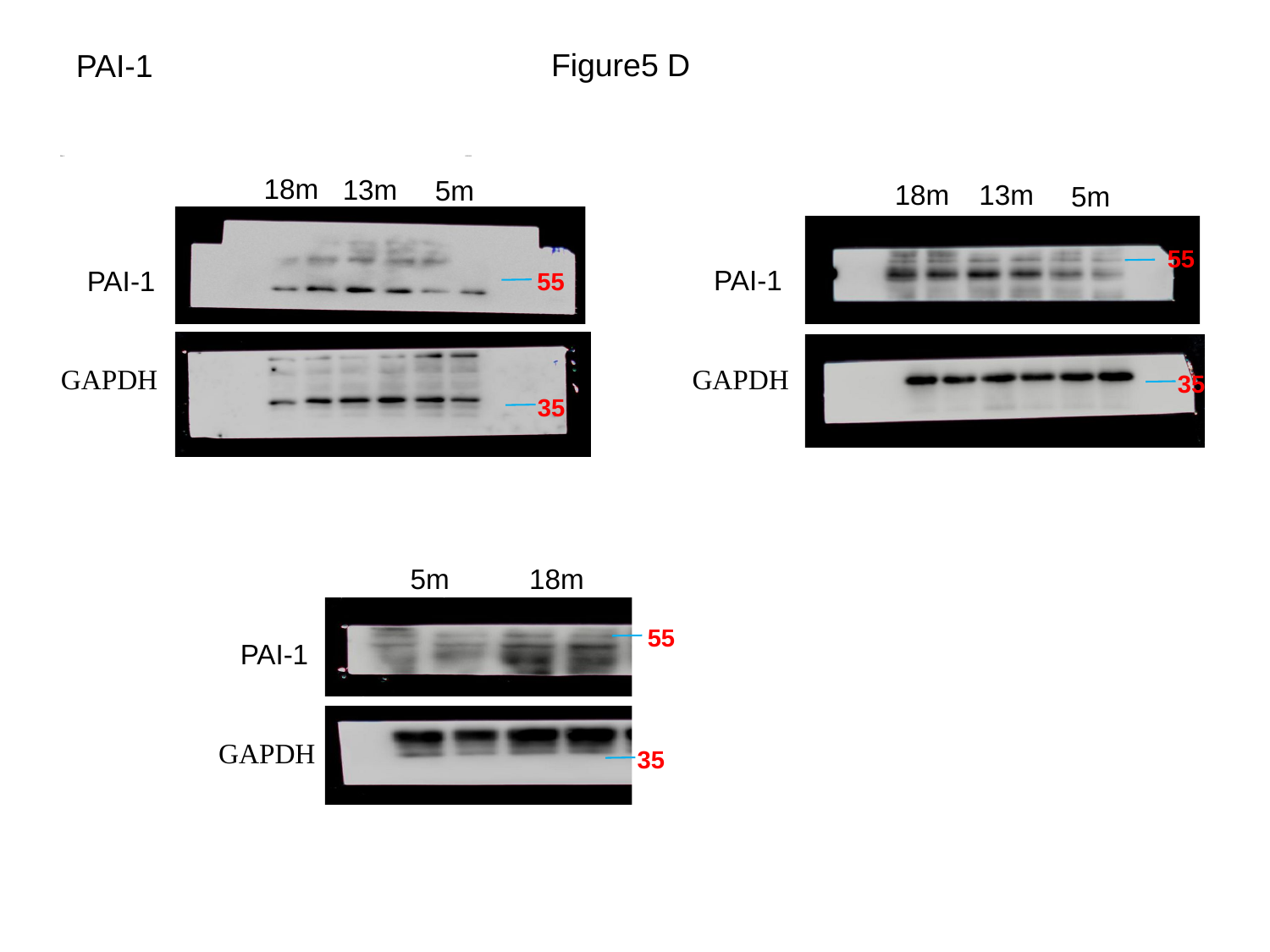

Figure5 D
PAI-1
PAI-1
不同月龄小鼠
18m
13m
5m
18m
13m
5m
55
PAI-1
PAI-1
55
GAPDH
GAPDH
35
35
5m
18m
55
PAI-1
GAPDH
35

## Slide 30
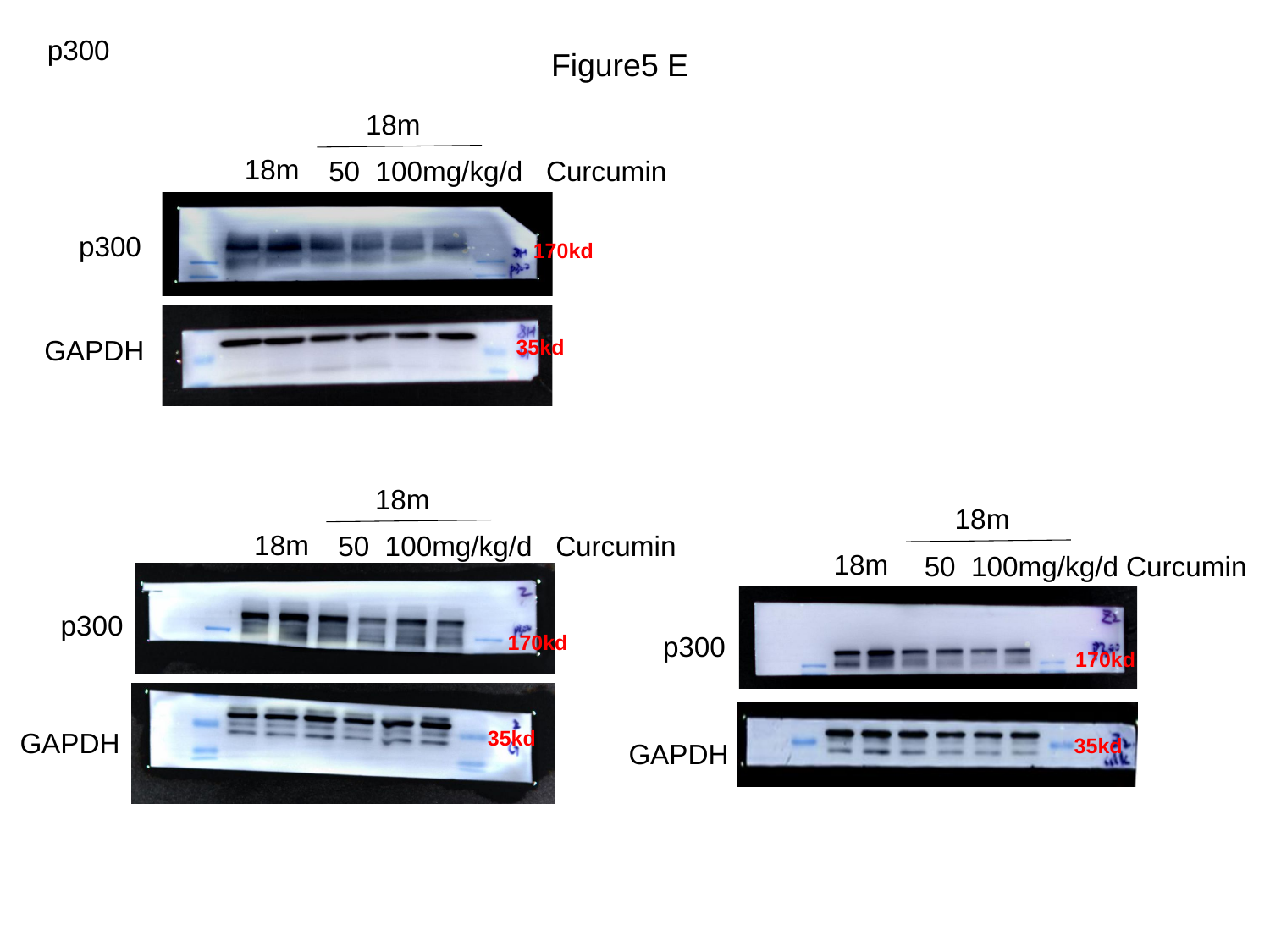

p300
Figure5 E
18m
18m
50 100mg/kg/d Curcumin
p300
170kd
GAPDH
35kd
18m
18m
18m
50 100mg/kg/d Curcumin
18m
50 100mg/kg/d Curcumin
p300
p300
170kd
170kd
35kd
GAPDH
35kd
GAPDH

## Slide 31
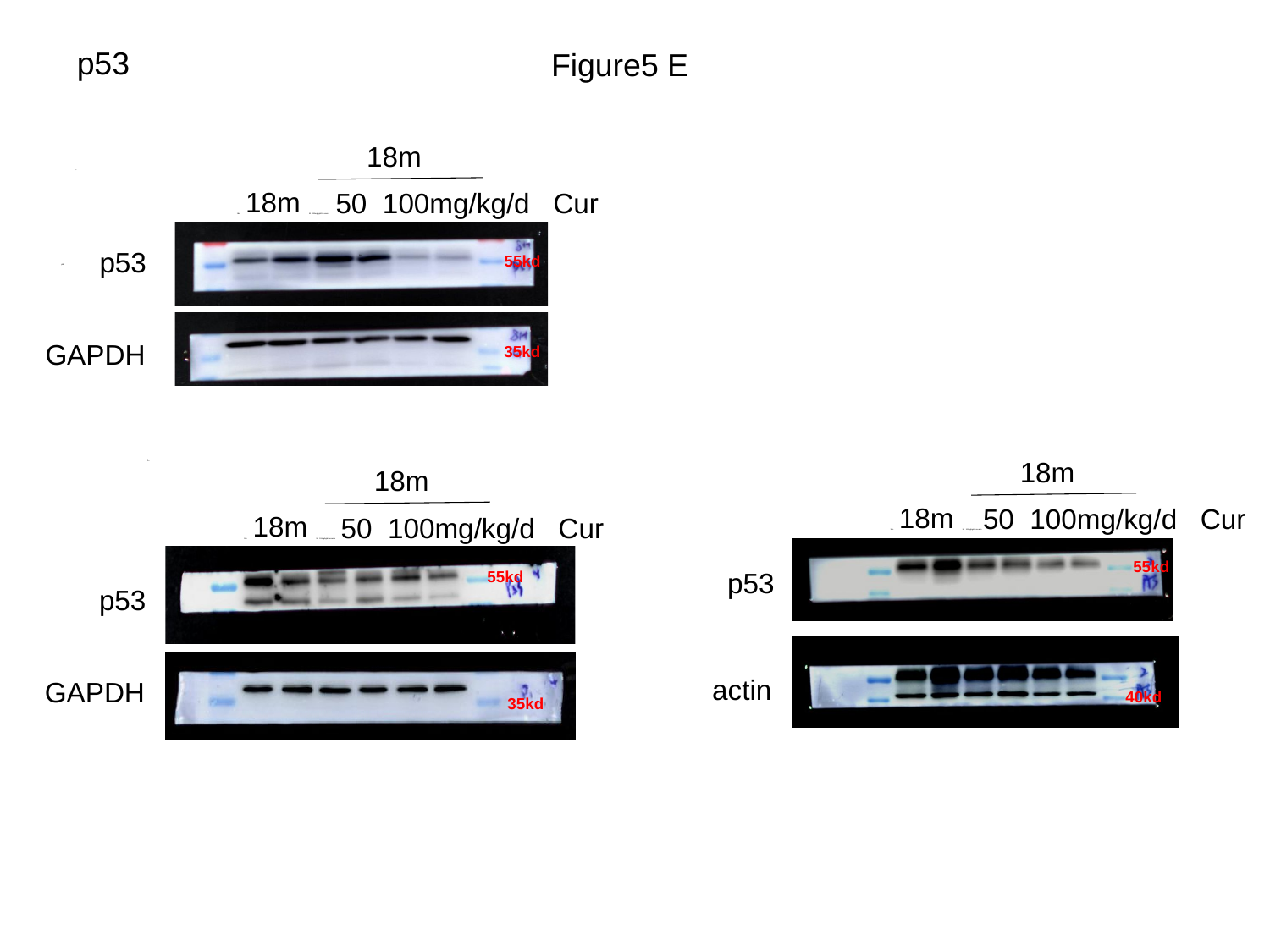

p53
Figure5 E
18m
3rd
18m
50 100mg/kg/d Cur
18m
50 100mg/kg/d Curcumin
50 100mg/kg/d Curcumin
p53
55kd
18m
p53
GAPDH
35kd
18m
2nd
18m
18m
50 100mg/kg/d Cur
18m
50 100mg/kg/d Cur
18m
50 100mg/kg/d Curcumin
18m
50 100mg/kg/d Curcumin
55kd
p53
55kd
p53
actin
GAPDH
40kd
35kd

## Slide 32
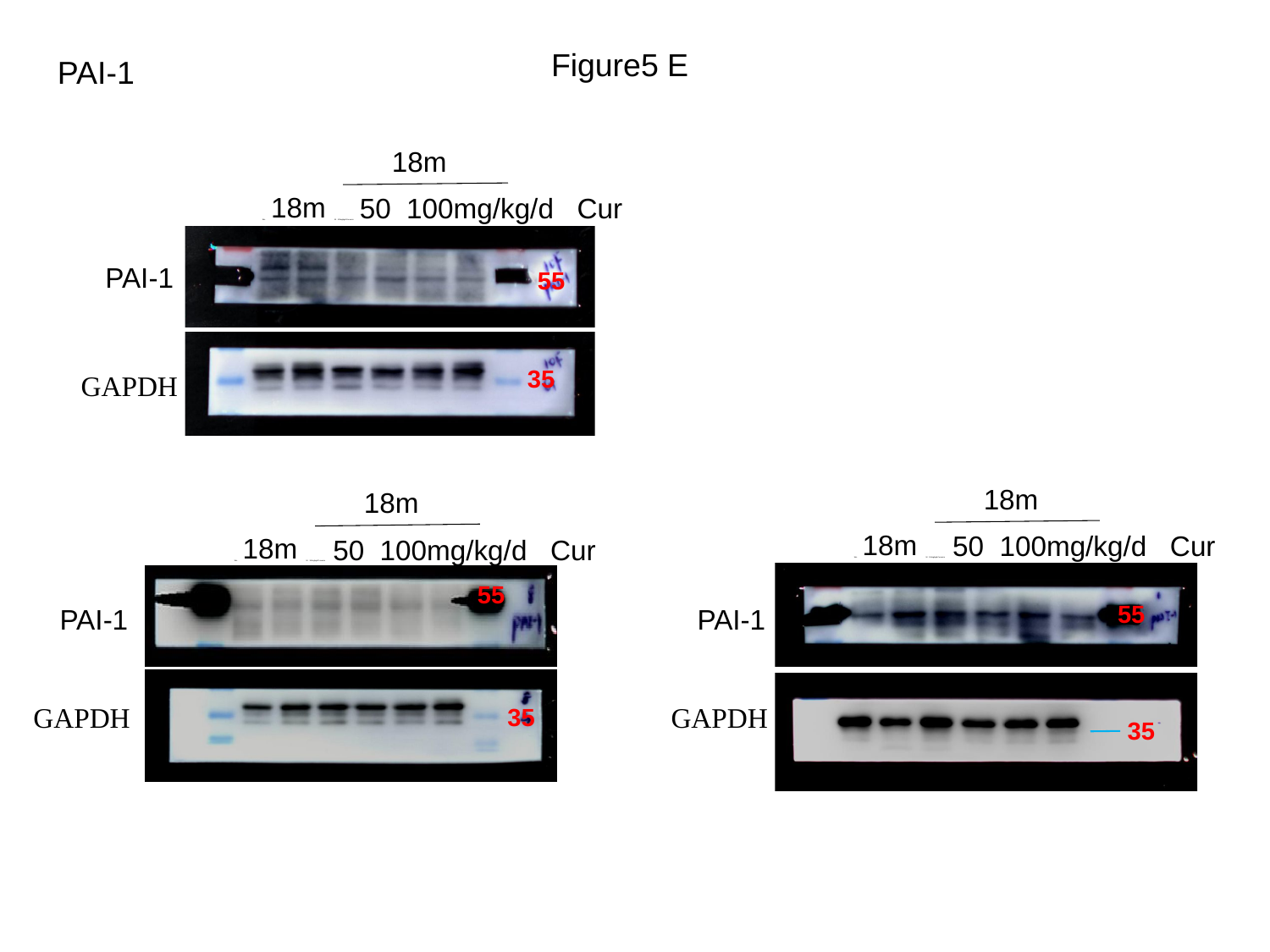

Figure5 E
PAI-1
18m
18m
50 100mg/kg/d Cur
18m
50 100mg/kg/d Curcumin
PAI-1
55
35
GAPDH
18m
18m
18m
50 100mg/kg/d Cur
18m
50 100mg/kg/d Cur
18m
50 100mg/kg/d Curcumin
18m
50 100mg/kg/d Curcumin
55
55
PAI-1
PAI-1
GAPDH
GAPDH
35
35

## Slide 33
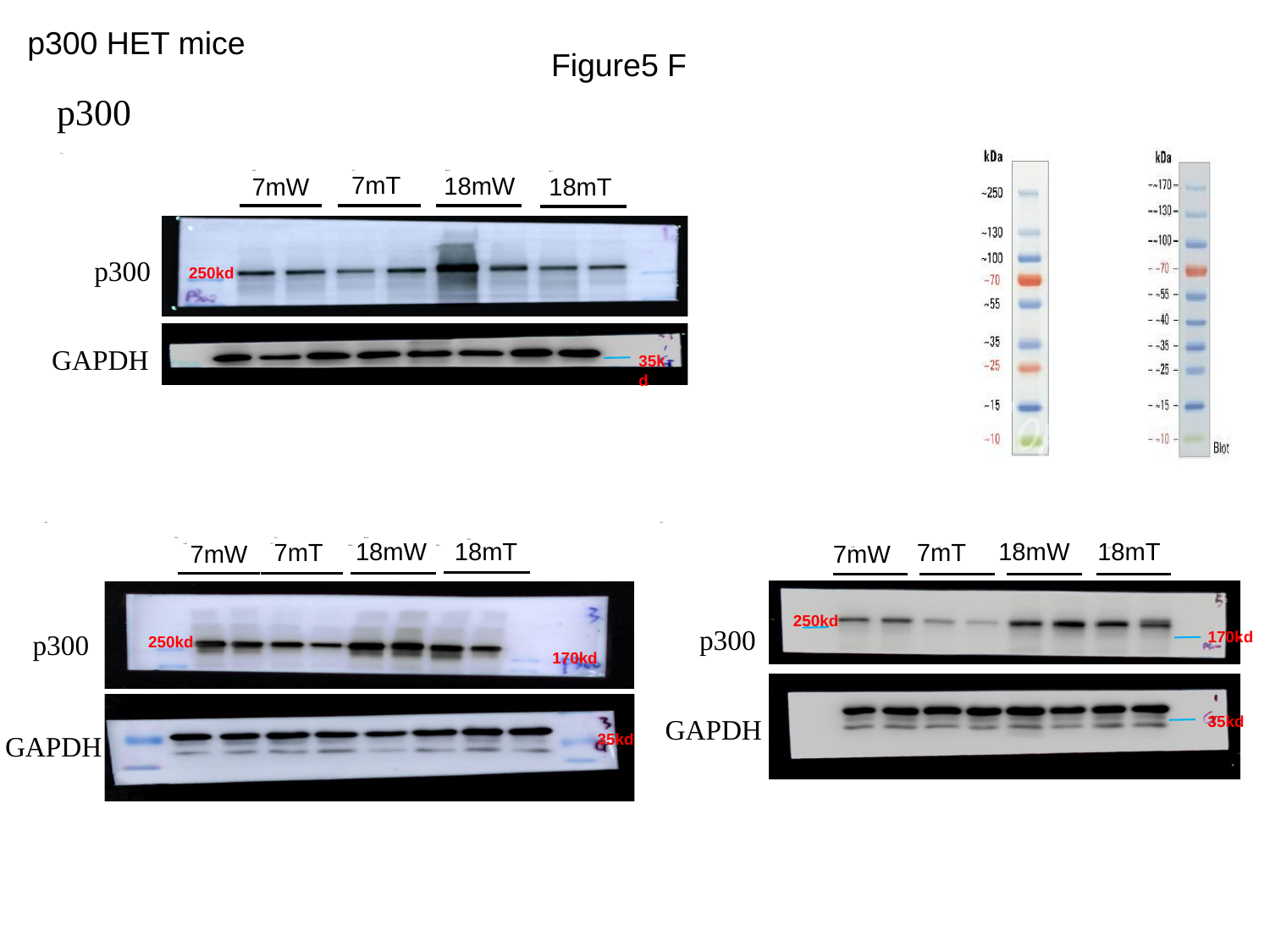

p300 HET mice
Figure5 F
p300
1st
7mT
7mW
7mT
18mW
18mW
7mW
18mT
18mT
p300
250kd
GAPDH
35kd
2nd
3rd
18mW
18mT
18mW
18mT
7mT
7mT
7mW
7mT
18mW
7mW
18mT
7mW
7mW
7mT
18mW
18mT
7mW
7mT
18mT
18mW
250kd
p300
p300
170kd
250kd
170kd
GAPDH
35kd
GAPDH
35kd

## Slide 34
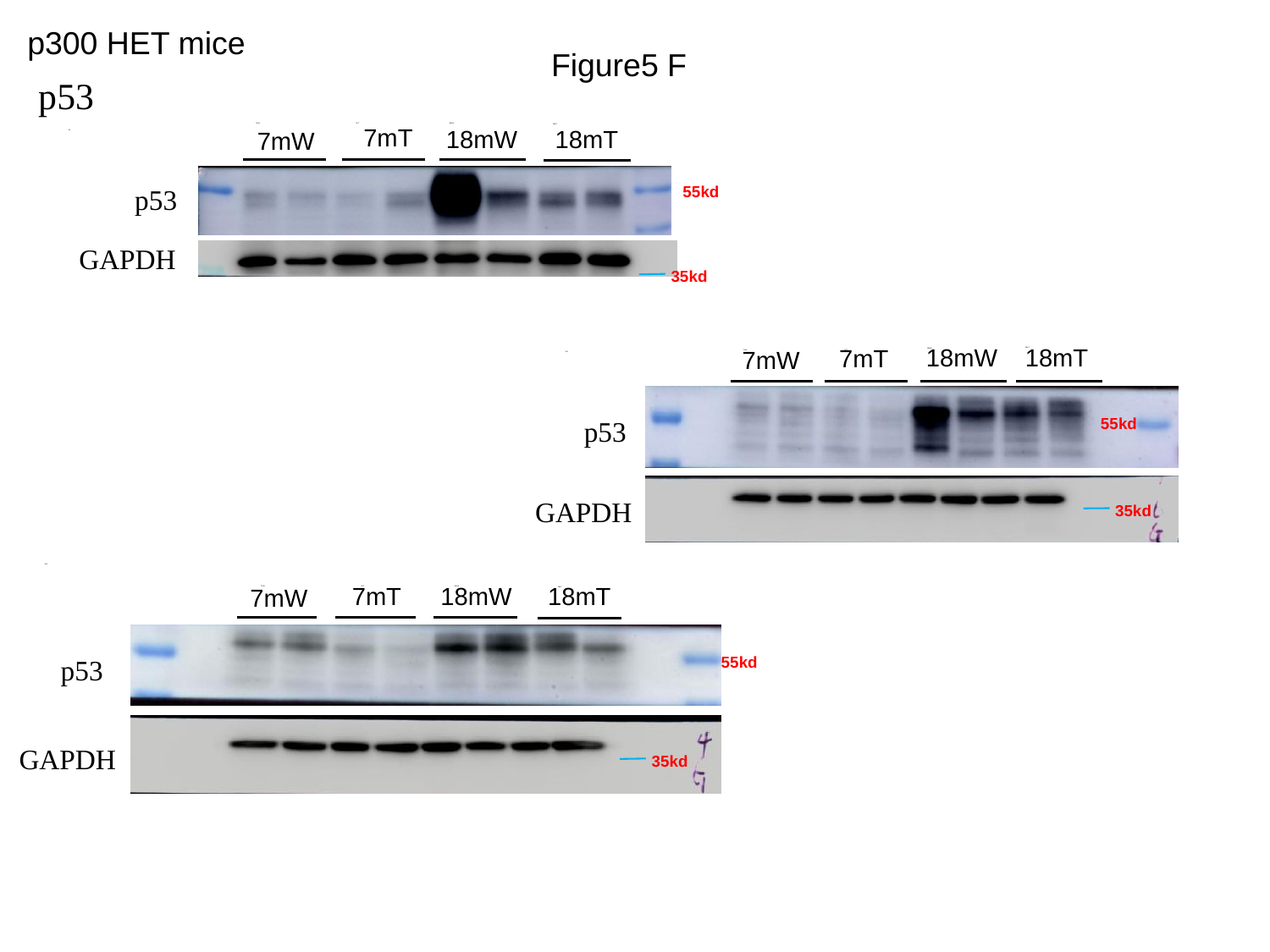

p300 HET mice
Figure5 F
p53
7mW
7mT
7mT
18mW
18mT
18mW
18mT
7mW
1st
p53
55kd
GAPDH
35kd
18mW
18mT
7mT
7mW
18mT
18mW
7mW
7mT
3rd
55kd
p53
GAPDH
35kd
2nd
18mW
18mT
7mT
7mW
7mW
7mT
18mW
18mT
p53
55kd
GAPDH
35kd

## Slide 35
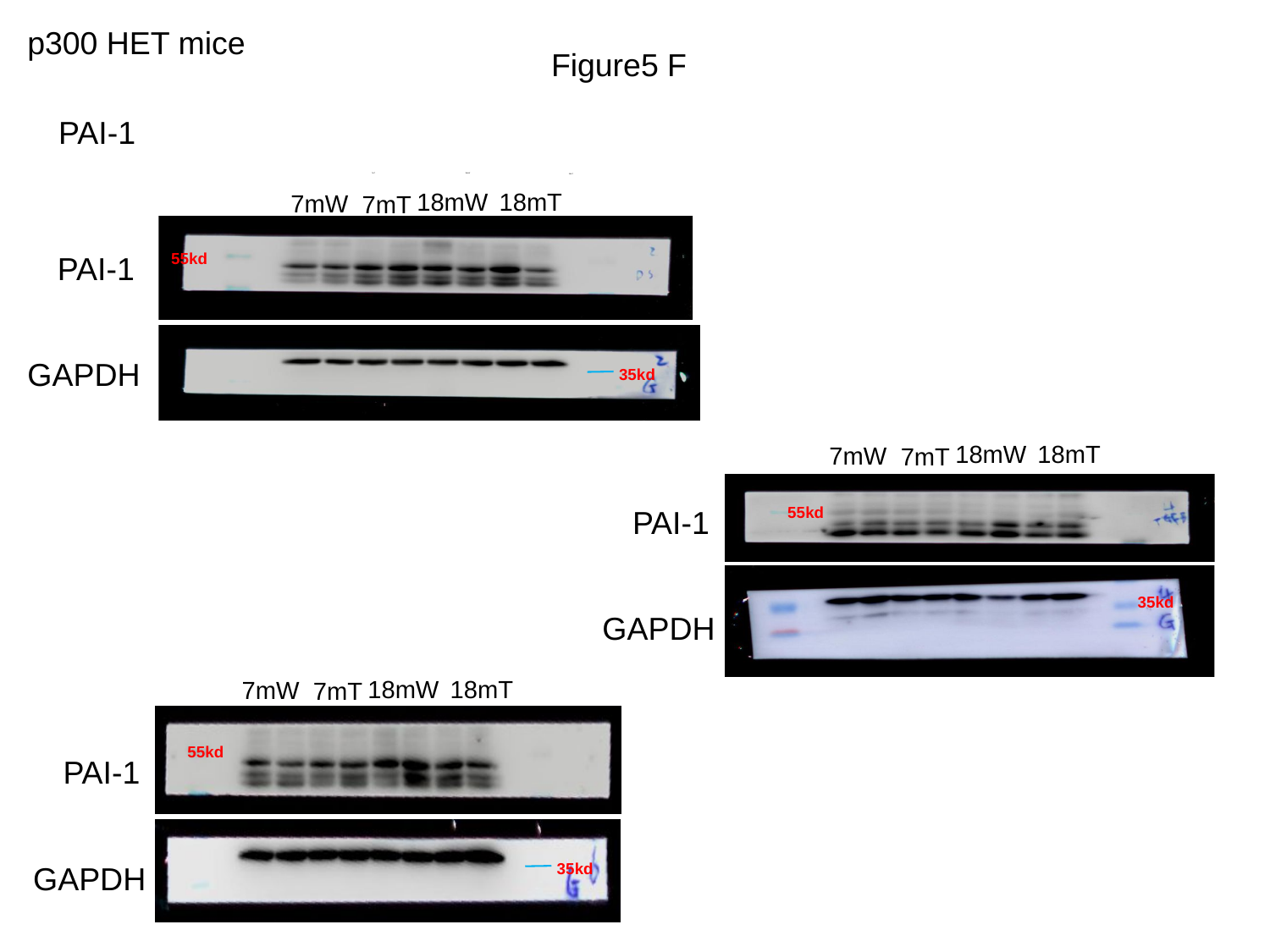

p300 HET mice
Figure5 F
PAI-1
7mT
18mW
18mT
18mW
18mT
7mW
7mT
PAI-1
55kd
GAPDH
35kd
18mW
18mT
7mW
7mT
PAI-1
55kd
35kd
GAPDH
18mW
18mT
7mW
7mT
55kd
PAI-1
GAPDH
35kd
